# Strategies for Designing Antithermal‐Quenching Red Phosphors

**DOI:** 10.1002/advs.201903060

**Published:** 2020-02-29

**Authors:** Yi Wei, Hang Yang, Zhiyu Gao, Yixin Liu, Gongcheng Xing, Peipei Dang, Abdulaziz A. Al Kheraif, Guogang Li, Jun Lin, Ru‐Shi Liu

**Affiliations:** ^1^ Engineering Research Center of Nano‐Geomaterials of Ministry of Education Faculty of Materials Science and Chemistry China University of Geosciences Wuhan Hubei 430074 P. R. China; ^2^ State Key Laboratory of Rare Earth Resource Utilization Changchun Institute of Applied Chemistry Chinese Academy of Sciences Changchun Jilin 130022 P. R. China; ^3^ Dental Health Department College of Applied Medical Sciences King Saud University Riyadh 12372‐3308 Saudi Arabia; ^4^ Department of Chemistry National Taiwan University Taipei 106 Taiwan; ^5^ Department of Mechanical Engineering and Graduate Institute of Manufacturing Technology National Taipei University of Technology Taipei 106 Taiwan

**Keywords:** antithermal quenching, color adjustment, Eu^3+^ → Mn^4+^ energy transfer, red phosphor, white light emitting diodes (w‐LEDs)

## Abstract

Nowadays, red phosphor plays a key role in improving the lighting quality and color rendering index of phosphor‐converted white light emitting diodes (w‐LEDs). However, the development of thermally stable and highly efficient red phosphor is still a pivotal challenge. Herein, a new strategy to design antithermal‐quenching red emission in Eu^3+^, Mn^4+^‐codoped phosphors is proposed. The photoluminescence intensity of Mg_3_Y_2(1−_ *_y_*_)_Ge_3_O_12_:*y*Eu^3+^, Mn^4+^ (0 ≤ *y* ≤ 1) phosphors continuously enhances with rising temperature from 298 to 523 K based on Eu^3+^ → Mn^4+^ energy transfer. For Mg_3_Eu_2_Ge_3_O_12_:Mn^4+^ sample, the integrated intensity at 523 K remarkably reaches 120% of that at 298 K. Interestingly, through codoping Eu^3+^ and Mn^4+^ in Mg_3_Y_2_Ge_3_O_12_, the photoluminescence color is controllably tuned from orangish‐red (610 nm) to deep‐red (660 nm) light by changing Eu^3+^ concentration. The fabricated w‐LEDs exhibit superior warm white light with low corrected color temperature (CCT = 4848 K) and high color rendering index (*R*_a_ = 96.2), indicating the promising red component for w‐LED applications. Based on the abnormal increase in antistokes peaks of Mn^4+^ with temperatures, Mg_3_Eu_2_Ge_3_O_12_:Mn^4+^ phosphor also presents a potential application in optical thermometry sensors. This work initiates a new insight to construct thermally stable and spectra‐tunable red phosphors for various optical applications.

## Graphical abstract

A proposal for designing antithermal‐quenching red phosphors with consecutive emission enhancement (beyond 120%) up to 523 K and controllably luminescence color adjustment from orange to deep red light based on Eu^3+^ → Mn^4+^ energy transfer for potential application in white light emitting diodes and optical low‐temperature thermometry sensors is presented.

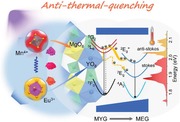

## 1 Introduction

Phosphor‐converted white light emitting diodes (w‐LEDs) have become the next‐generation lighting source due to high efficiency, low‐energy consumption, long lifetime, and environmental compatibility, and so on.^1^, ^2^, ^3^ The common w‐LEDs devices are fabricated via two combination strategies: 1) blue LED chip and yellow phosphor; 2) near‐ultraviolet (n‐UV) LED chip and tricolor phosphors.^4^, ^5^ No matter for which fabrication methods, the development of red phosphor is crucial to improve the lighting quality and tune corrected color temperature of w‐LEDs.^6^, ^7^ To date, many researchers have focused on exploring highly efficient red phosphors. Although Eu^2+^‐doped nitride phosphors such as CaAlSiN_3_:Eu^2+^ and Sr_2_Si_5_N_8_:Eu^2+^ 8, 9, 10 show high quantum yield (QY > 90%) and high thermal quenching temperature (>600 K), the harsh preparation conditions (high pressure ≥ 0.9–2.5 MPa; high temperature ≥1700–200 °C) and deep‐red emission position (beyond 650 nm) limit the large‐scale application in indoor lighting. Eu^3+^‐doped inorganic compounds are typical red‐emitting phosphors due to the (4f^6^)^5^D_0_ → (4f^6^)^7^F_J_ spin‐ and parity‐forbidden transition,^11^, ^12^ but it is hardly utilized in w‐LEDs applications owing to the linearly narrow excitation and emission.^13^, ^14^ Mn^4+^ has been considered as the promising red‐emitting activator ions, because Mn^4+^‐activated fluoride phosphors successfully achieve narrow‐band red emission (emission peaks locate at 600–700 nm) and high QY > 98%.^15^, ^16^, ^17^ Unfortunately, the use of toxic fluohydric acid and terribly chemical and environmental stability limit the commercial application in w‐LEDs. Recently, the emerging Mn^4+^‐doped oxide phosphors attract much attention owing to the facile synthesis method and highly environmental stability. Regrettably, their luminescence quantum yield is low (QY < 70%), and it presents a deep‐red emission (660–750 nm), which usually exceeds the sensitivity of people's eyes (around 650 nm).^18^, ^19^ Therefore, the optimization of current red phosphors with high efficiency and spectral tunability is still a crucial challenge.

Usually, thermal generation (>473 K) during w‐LEDs operation easily results in serious emission loss due to the nonradiative relaxation. That is so‐called thermal quenching,^20^ which is a vital index to evaluate practical application of phosphors in w‐LEDs. Recently, Qiao et al. systematically summarized the strategies to decrease the thermal quenching performance of phosphors, including defect levels, cation disorder, structural rigidity, phosphors coating and phosphors in glass. These designing methods can help to solve the thermal quenching behavior in many phosphor materials.^21^ The main reason is that thermal quenching property is strongly associated with the crystal structure such as structure rigidity and symmetry. The matrixes (such as garnets, phosphates, and nitrides) with dense framework usually generates a low thermal quenching behavior.^22^ Previously, many studies dedicated to exploring blue and green phosphors with ultralow thermal quenching performance. Kim et al. first reported a zero‐thermal‐quenching blue Na_3_Sc_2_(PO_4_)_3_:Eu^2+^ phosphor that is caused by the phase‐transition‐induced defect formation.^23^ Zhao et al. explored green‐emitting RbLi(Li_3_SiO_4_)_2_:Eu^2+^ phosphor with very low thermal quenching (103%@150 °C of the integrated photoluminescence intensity at 20 °C) due to the strong lattice rigidity.^24^ However, low‐thermal‐quenching red phosphors were rarely reported, easily leading to the low color rendering index and white imbalance for w‐LEDs lighting.^25^, ^26^, ^27^ Consequently, the serious thermal quenching behavior of red‐emitting phosphor is another urgent challenge for its practical application in w‐LEDs lighting.

To the best of our knowledge, the garnet‐typed structures have been classified as one of the suitable phosphor host materials.^28^ In a typical A_3_B_2_C_3_O_12_ (A = alkali metal ions; B = alkaline earth metal ions; C = IVA metal ions) garnet structure, A, B, and C occupy the dodecahedral, octahedral, and tetrahedral sites, respectively.^29^, ^30^ Owing to the dense framework and symmetric lattice structure, garnet structures can accommodate rare‐earth ions and Mn^4+^ ions to realize high efficiency and stable photoluminescence.^4^ Using garnet structure as a template, in the present work, we propose a novel strategy to develop red Eu^3+^, Mn^4+^‐codoped Mg_3_Y_2_Ge_3_O_12_ (MYG) phosphor that possesses antithermal‐quenching property as well as the color adjustability. This is the first time to report the antithermal‐quenching behavior from the Eu^3+^ → Mn^4+^ energy transfer strategy, which opens a new gateway to obtain thermally stable luminescence materials. In addition, these phosphors can act as a superb red candidate in warm w‐LEDs devices and optical thermometry sensors. Therefore, this discovery opens a door to develop antithermal‐quenching property and high luminescence efficiency in Mn^4+^‐contained oxide phosphors.

## 2 Results and Discussion

**Figure 1:**
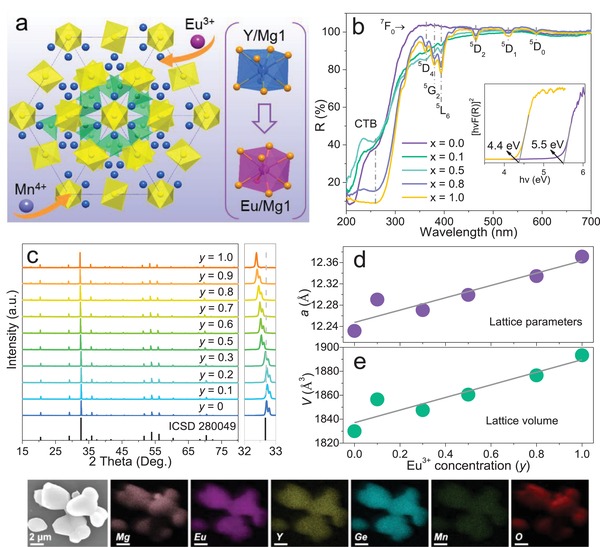
a) Crystal structure of MYG along [111] direction, where blue, violet, yellow, green, and orange spheres represent Y, Eu, Mg, Ge, O atoms respectively. b) Diffuse reflectance spectra of MYG:*x*Eu^3+^ (0 ≤ *x* ≤ 1), the inset shows the optical bandgap values for MYG and MEG matrixes. c) XRD patterns of MYG:*y*Eu^3+^, 0.01Mn^4+^ (0 ≤ *y* ≤ 1). d,e) Lattice parameter *a* and volume *V* versus Eu^3+^ concentration in MYG:*y*Eu^3+^, 0.01Mn^4+^ (0 ≤ *y* ≤ 1). f) SEM images and elemental mapping analysis for MYG:0.6Eu^3+^, 0.01Mn^4+^ (The scalebar is 2 µm).

MYG belongs to cubic Ia‐3d phase. There are three types of polyhedra that connect each other by sharing a corner and edge (**Figure** **1**a). The dodecahedral sites are occupied by two‐third Y^3+^ and one‐third Mg^2+^ ions at 24c sites, the octahedral sites are totally occupied by Mg^2+^ ions at 16a sites, and tetrahedral sites are occupied by Ge^4+^ ions at 24d sites. Previously, some articles reported the successful incorporation of rare earth Ce^3+^/Eu^3+^ ions and non‐rare earth Bi^3+^ ions in MYG matrix,^31^, ^32^, ^33^ these results demonstrate that MYG is suitable as phosphor matrix. Due to the similar ionic radii between Eu^3+^ (CN = 8, *r* = 1.066 Å; CN represents coordination number, *r* is ion radius) and Y^3+^ (CN = 8, *r* = 1.019 Å), Eu^3+^ is designed to substitute Y^3+^ until a complete cantonment. All diffraction peaks in XRD patterns match well with the standard MYG (ICSD No. 280049) phase (Figure S1, Supporting Information). The (420) lattice plane gradually shifts to a small angle direction at 2θ = 32–33°, confirming the successful incorporation of Eu^3+^ ions. Rietveld refinement of XRD pattern is performed to characterize the microstructure evolution, the accredited R‐factors of MYG:*x*Eu^3+^ (0 ≤ *x* ≤ 1) indicate the formation of solid solution phase (Figure S2a–f and Table S1, Supporting Information). Since Eu^3+^ is slightly bigger than that of Y^3+^, the lattice parameter *a* and volume *V* linearly increase (Table S1 and Figure S3, Supporting Information), which obeys the Vegard law.

Diffuse reflectance spectra of MYG:*x*Eu^3+^ (0 ≤ *x* ≤ 1) were measured to clarify the electron configuration evolution between MYG and MEG. Figure 1b displays the characteristic Eu^3+^ absorption band in the n‐UV and blue region with the maximum at 393 nm (^7^F_0_ → ^5^L_6_). All peaks intensities gradually increase with increasing Eu^3+^ concentration, which is beneficial to the practical application in w‐LEDs. The calculated bandgap values are 5.5, 4.4 eV for MYG and MEG (Inset of Figure 1b), respectively. The decreasing bandgap indicates that the incorporation of Eu^3+^ generates electron rearrangement in MYG matrix because of the different electronegativity between Eu^3+^ and Y^3+^. Except for acting as a matrix framework, Eu^3+^ also emits orangish‐red light in MYG:*x*Eu^3+^ (0 ≤ *x* ≤ 1). The photoluminescence excitation (PLE) spectra depict a strong excitation in the n‐UV region with a maximum at 394 nm, in good consistency with the results of diffuse reflectance spectra (Figure S4a,b, Supporting Information). A redshift of O^2−^ → Eu^3+^ charge transition band (CTB) appears with increasing Eu^3+^ concentration. Generally, the bigger M^3+^ metal ions will generate a weaker potential energy to O^2−^ ions,^34^, ^35^ thus, the gradual substitution of larger Eu^3+^ ions for Y^3+^ is the main reason for the redshift of CTB. This phenomenon provides an evidence for the local electron configuration variation. Under 394 nm excitation, the optimal Eu^3+^ doping level is 0.4, the quenching mechanism is multipolar interactions (Figure S4c, Supporting Information). With increasing Eu^3+^ concentration, the decreasing peak intensity ratio *I*_590_/*I*_613_ and increasing distortion index suggest the decreasing lattice symmetry (Figure S4d,f, Supporting Information). The above results indicate that MYG:*x*Eu^3+^ (0 ≤ *x* ≤ 1) solid solution phases offer abundant lattice environment for activators. Because the deep‐red emission of MYG:Mn^4+^ phosphor is not suitable for w‐LEDs applications,^36^ Mn^4+^ ions were incorporated into MYG:*x*Eu^3+^ (0 ≤ *x* ≤ 1) to design energy transfer and achieve photoluminescence adjustment. All MYG:*y*Eu^3+^, 0.01Mn^4+^ (0 ≤ *y* ≤ 1) phosphors are pure phases (Figure 1c). The linear shift of the enlarged XRD pattern (2θ = 32–33°) confirms the simultaneous substitution of Mn^4+^ → Mg^2+^ and Eu^3+^ → Y^3+^. In Figure S5 in the Supporting Information, the Rietveld refinement results and the lattice parameters (*a* and *V*) of MYG:*y*Eu^3+^, 0.01Mn^4+^ (0 ≤ *y* ≤ 1) further prove the successful introduction of Mn^4+^ and Eu^3+^. Figure 1f depicts the morphology and elemental distribution of the representative MYG:0.6Eu^3+^, 0.01Mn^4+^, which displays slightly agglomerate spheres with a diameter of 1–4 µm. The smooth surface clarifies the high crystallinity degree. The elemental mapping images give a direct evidence that Mg, Eu, Y, Ge, Mn, O homogeneously distribute in MYG:0.6Eu^3+^, 0.01Mn^4+^. Furthermore, the scanning electron microscope‐energy‐dispersive X‐ray spectrum (SEM‐EDS) analysis of MYG:0.6Eu^3+^, 0.01Mn^4+^ sample demonstrates the average composition (15 points of different positions) of Mg:Y:Eu:Ge:Mn:O equals to 3.98:0.99:1.30:3:0.05:10.53, which is close to the theoretical ratio of 3:0.8:1.20:3:0.03:12. The above results indicate the successful incorporation of Mn^4+^ and Eu^3+^.

**Figure 2:**
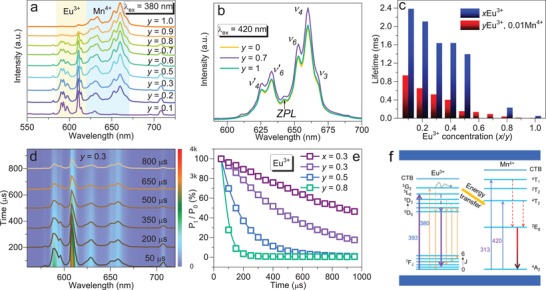
a) Normalized PL spectra of MYG:*y*Eu^3+^, 0.01Mn^4+^ (0.1 ≤ *y* ≤ 1) (λ_ex_ = 380 nm). b) PL spectra of MYG:*y*Eu^3+^, 0.01Mn^4+^ (*y* = 0, 0.7, 1) (λ_ex_ = 420 nm). c) The PL decay lifetimes of Eu^3+^ in MYG:*x*Eu^3+^ and MYG:*y*Eu^3+^, 0.01Mn^4+^ (0.1 ≤ *x*/*y* ≤ 1) (λ_ex_ = 393 nm, λ_em_ = 610 nm). d) The time‐resolved PL spectra and the representative PL spectra for MYG:0.3Eu^3+^, 0.01Mn^4+^. e) The peak intensity ratio (*P*_t_/*P*_0_) versus time in MYG:0.3Eu^3+^ and MYG:*y*Eu^3+^, 0.01Mn^4+^ (*y* = 0.3, 0.5, 0.8). f) Schematic Eu^3+^ → Mn^4+^ energy transfer diagram.

As expected, a series of color‐tunable red phosphors are obtained. Both diffuse reflectance and PLE spectra demonstrate that the absorption region locates from 250 to 475 nm (Figure S6, Supporting Information). Upon 380 nm irradiation, continuous photoluminescence adjustment from orangish‐red to deep‐red light appears in MYG:*y*Eu^3+^, 0.01Mn^4+^ (0.1 ≤ *y* ≤ 1) (**Figure** **2**a; Figure S7, Supporting Information). At *y* = 0.1, the samples only present orangish‐red emission at 610 nm (^5^D_0_ → ^7^F_1_ of Eu^3+^). At 0.1 ≤ *y* ≤ 0.5, the emission peaks in the deep‐red region at 633 and 660 nm (^2^E_g_ → ^4^A_2_ of Mn^4+^) appear and the emission intensity gets stronger. At *y* = 0.5, the peak intensity of Eu^3+^ almost equals to Mn^4+^. At 0.5 ≤ *y* ≤ 1, the emission intensity of Eu^3+^ is weaker than that of Mn^4+^. The photoluminescence emission (PL) spectra measured at 393 nm also exhibit a similar photoluminescence adjustment (Figure S8, Supporting Information). Especially, MEG:0.01Mn^4+^ sample completely displays typical Mn^4+^ emission at 660 nm (Figure S9, Supporting Information), further proving the Eu^3+^ → Mn^4+^ energy transfer. At an optimal excitation wavelength of Mn^4+^, several narrow bands appear with the strongest emission locating at 660 nm (Figure 2b). The 642 nm peak is typical zero photon line (ZPL) peak. The high‐energy peaks (626, 633 nm) belong to antistokes ν′_4_, and ν′_6_, and the low‐energy peaks (668, 660, and 653 nm) are assigned to stokes ν_3_, ν_4_, and ν_6_,^37^ respectively. The PL intensity of Eu^3+^, Mn^4+^‐codoped MYG is stronger than that of MYG:0.01Mn^4+^, confirming the occurrence of energy transfer from Eu^3+^ to Mn^4+^. Besides, under 393 nm excitation at room temperature, the internal quantum yield enhances from 15.1% to 76.5% for MYG:0.01Mn^4+^ and MYG:0.3Eu^3+^, 0.01Mn^4+^ in the region of 380–800 nm, demonstrating the energy transfer plays a key role in improving lighting quality of Mn^4+^. As shown in Figure S10 in the Supporting Information, after exposing in air for 60 h, the profiles and shapes of PL spectra for MYG:0.6Eu^3+^, 0.01Mn^4+^ sample remain unchanged under 380, 393, 420 nm wavelengths excitation. The integrated and peak intensities (610 and 660 nm) after 60 h can keep at least 86.5% of the original intensity (Figure S10d, Supporting Information). All the above investigations indicate the stable luminescence and energy transfer in air.

The PL decay curves of Eu^3+^ were measured to clarify the energy transfer with λ_ex_ = 393 nm and λ_em_ = 610 nm (Figure 2c; Figure S11, Supporting Information). The lifetimes of MYG:*x*Eu^3+^ (0.1 ≤ *x* ≤ 1) are calculated to be 0.051–2.382 ms, which is higher than that of MYG:*y*Eu^3+^, 0.01Mn^4+^ (0.1 ≤ *y* ≤ 1) (0.009–0.935 ms). This result proves the highly efficient energy transfer from Eu^3+^ to Mn^4+^. The energy transfer efficiency values are calculated to be 60.8%, 69%, 68.3%, 75.4%, 88.9%, 83.1%, and 82.6% for 0.1 ≤ *y* ≤ 1 (Figure S12a, Supporting Information), respectively. The above results indicate that the energy transfer efficiency gradually increases with increasing Eu^3+^ concentration. In addition, the critical distance (*R*_c_) equals to 11.4 Å based on the formula (1) in supporting information, demonstrating that the energy transfer is mainly attributed to the multipolar interaction. According to Dexter's energy transfer formula^38^, ^39^

(1) $$\frac{I_{S0}}{I_{S}}\;\propto \;C^{\frac{\theta }{3}}$$

in which *I*_S0_ and *I*_S_ represent the emission intensity without and with Mn^4+^, C is the sum of Eu^3+^ and Mn^4+^ concentration. The values of θ = 6, 8, 10 correspond to dipole–dipole, dipole—quadrupole, and quadrupole–quadrupole interactions, respectively. Figure S12b–d in the Supporting Information plot linear relationship of *I*_S0_/*I*_S_ − C*^θ^* ^/3^, The maximal correlation coefficient of the linear fitting can be achieved at θ = 6, indicating that the dipole–dipole interaction mainly contributes to the Eu^3+^ → Mn^4+^ energy transfer in MYG:*y*Eu^3+^, 0.01Mn^4+^ (0.1 ≤ *y* ≤ 1) samples. In addition, time‐resolved PL spectra can accurately analyze the dynamic process of energy transfer.^40^ All PL intensities of MYG:*y*Eu^3+^, 0.01Mn^4+^ (0.1 ≤ *y* ≤ 1) samples gradually decrease with irradiation time. After continuously irradiating 800 µs, the PL intensity of Eu^3+^ remains 54% in MYG:0.3Eu^3+^ (Figure S13a, Supporting Information). While the PL intensity only keeps 23% in MYG:0.3Eu^3+^, 0.01Mn^4+^ sample (Figure 2d), and the PL decay rate gets faster with increasing Eu^3+^ concentration, even the PL intensity disappears at 600 and 200 µs for *y* = 0.5 and 0.8, respectively (Figure 2e; Figure S13b,c, Supporting Information). Inversely, the PL decay rate of Mn^4+^ gets slower (Figures S13d and S14, Supporting Information), whose PL intensity remains 50%, 67%, 63%, and 67% for *y* = 0, 0.3, 0.5, and 0.8, respectively. The phenomena offer a powerful evidence for Eu^3+^ → Mn^4+^ energy transfer. A schematic energy transfer diagram is proposed in Figure 2f. Under n‐UV illumination, the electron in the ground energy level of Eu^3+^ is excited to the excited energy levels, then the excited electron will release energy in the following two pathways: On one hand, the excited electron relaxes to ^5^D_0_ energy level, and returns back to the ground energy level with 610 nm orangish‐red light. On the other hand, the energy in excited energy levels of Eu^3+^ can transfer to ^2^T_2_, ^4^T_2_ energy levels of Mn^4+^. Compared with the ^5^L_6_, the highest ^5^G_2_ energy level easily transfers energy to ^2^T_2_, ^4^T_2_ energy levels of Mn^4+^ rather relaxes back to the lowest ^5^D_0_ excited energy level, as a result, the Eu^3+^ → Mn^4+^ energy transfer occurs.

**Figure 3:**
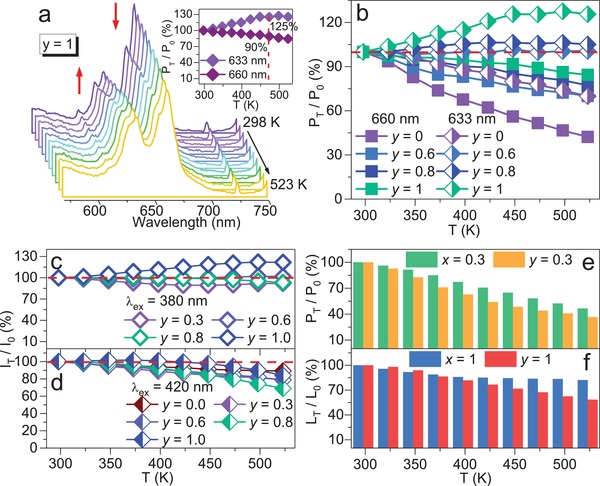
a) Temperature‐dependent PL spectra of MEG: 0.01Mn^4+^ (*y* = 1) at 298–523 K (λ_ex_ = 380 nm), the inset plots the relative peak intensity ratio (*P*_t_/*P*_0_) versus temperature. b) *P*_t_/*P*_0_ of Mn^4+^ versus temperature in MYG:*y*Eu^3+^, 0.01Mn^4+^ (0 ≤ *y* ≤ 1, λ_ex_ = 380 nm). Relative integrated intensity ratio (*I*_T_/*I*_0_) versus temperature in MYG:*y*Eu^3+^, 0.01Mn^4+^ (0 ≤ *y* ≤ 1) monitored at c) λ_ex_ = 380 nm and d) λ_ex_ = 420 nm. e) Relative peak intensity ratio (*P*_t_/*P*_0_) of Eu^3+^ versus temperature in MYG:*y*Eu^3+^, 0.01Mn^4+^ (0 ≤ *y* ≤ 1, λ_ex_ = 380 nm). f) Relative lifetime ratio (*L*_T_/*L*_0_) of Eu^3+^ versus heating temperature for MEG and MEG:0.01Mn^4+^ samples (λ_ex_ = 380 nm, λ_em_ = 610 nm).

Antithermal‐quenching behavior is urgently expected for red phosphors to achieve high‐quality lighting of w‐LEDs.^4^ The thermal quenching behavior appears in MYG:*x*Eu^3+^ (0.1 ≤ *x* ≤ 1), the PL intensity at 473 K keeps 80%‐92% of that at room temperature. With increasing Eu^3+^, the thermal quenching property gets lower, which is attributed to the increasing active energy (Figure S15, Supporting Information). For MEG:0.01Mn^4+^ sample (*y* = 1), Mn^4+^ stokes *v*_4_ peak only decreases a little, whose P_473 K_/*P*_0_ (P_473 K_, *P*_0_ represent peak intensity at 473 and 298 K, respectively) value keeps as high as 90%. Unexpectedly, the Mn^4+^ antistokes *v*_6_′ peak gradually increases with P_473 K_/*P*_0_ reaching 125% (**Figure** **3**a). Actually, thermal quenching property of Mn^4+^ occurs in low Eu^3+^ concentration and gets smaller with increasing Eu^3+^ concentration from the temperature‐dependent PL spectra of MYG:*y*Eu^3+^, 0.01Mn^4+^ (0 ≤ *y* ≤ 1) (Figure S16, Supporting Information). As shown in Figure 3b, the P_473 K_/*P*_0_ value increases from 51% to 90% for stokes *v*_4_ peak, and from 78% to 125% for antistokes *v*_6_′ peak. Surprisingly, under 380 nm excitation, the relative integrated intensity ratio *I*_523 K_/*I*_0_ (*I*_T_ and *I*_0_ represent integrated intensity at 523 and 298 K, respectively) even reaches 120% at *y* = 1 (Figure 3c). Antithermal‐quenching and zero‐thermal‐quenching performances of MYG:*y*Eu^3+^, 0.01Mn^4+^ (0 ≤ *y* ≤ 1) also exhibit a similar tendency under 393 and 420 nm irradiation (Figure 3d; Figure S17, Supporting Information). The above results suggest that the antithermal‐quenching properties may be caused by Eu^3+^ → Mn^4+^ energy transfer. In addition, the long‐term photoluminescence stability of MYG:0.6Eu^3+^, 0.01Mn^4+^, and MEG:0.01Mn^4+^ samples at 373 and 423 K were analyzed. It is noted that the shape and intensity of MYG:0.6Eu^3+^, 0.01Mn^4+^ are almost the same with continuously heating at 423 K for 60 min (λ_ex_ = 380 nm) (Figure S18a, Supporting Information), demonstrating the ultrastable photoluminescence at high temperature. Compared with the initial intensity at room temperature, the integrated and peak intensities for MYG:0.6Eu^3+^, 0.01Mn^4+^, and MEG:0.01Mn^4+^ samples can stably keep antithermal‐quenching behavior for 60 min (Figure S18b–d, Supporting Information), further confirming the superior stability. Notably, it is the first time to obtain antithermal‐quenching performance based on the energy transfer strategy. To explain the influence of Eu^3+^ → Mn^4+^ energy transfer on the antithermal‐quenching behavior, the thermal quenching performance of Eu^3+^ is measured, which shows more serious thermal quenching in MYG:0.3Eu^3+^, 0.01Mn^4+^ than in MYG:0.3Eu^3+^ (Figure 3e). The L_473 K_ /*L*_0_ (L_473 K_ and *L*_0_ represent PL decay lifetime at 473 and 298 K, respectively) of Eu^3+^ equals to 84% and 67% for MEG and MEG:0.01Mn^4+^ (Figure 3f), respectively. This result indicates that the PL decay rate of Eu^3+^ gets much faster with codoping Mn^4+^, indicating that the Eu^3+^ → Mn^4+^ energy transfer happens.

**Figure 4:**
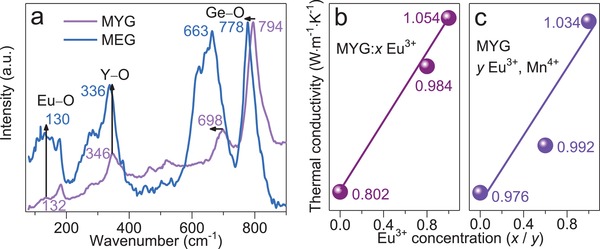
a) Raman spectra of MYG and MEG. The thermal conductivity versus Eu^3+^ concentration in b) MYG:*x*Eu^3+^ (*x* = 0, 0.8, 1) and c) MYG:*y*Eu^3+^, 0.01Mn^4+^ (*y* = 0, 0.6, 1).

**Figure** **4**a depicts Raman spectra of MYG and MEG matrixes, no new vibration bands appear, indicating the isostructural structure. With larger Eu^3+^ replacing Y^3+^, all vibration bands shift to low frequency, which is ascribed to the successful incorporation of heavy Eu^3+^ ions. The peak at around 130 cm^−1^ belongs to Eu–O vibration model, it gets stronger in the MEG matrix. The stretching vibration of Y—O bond shifts from 346 to 336 cm^−1^ with Eu^3+^ substituting Y^3+^.^41^ Moreover, the peaks at 794 and 698 cm^−1^ are assigned to Ge–O vibration.^42^ Accordingly, the highest‐energy phonons (*v*_max_) usually contribute to the relaxation process in the multi‐photon emitting system. For Mn^4+^ ions, the photon numbers (*n*) that overcome the nonradiative barrier can be expressed as *n* = ∆*E*_1_/*hv*_max_, ∆*E*_1_ is the energy gap between the emitting state and the next lower energy state. It is noted that the required photon number increases as the *v*_max_ value decreases from 794 to 778 cm^−1^ in MYG‐MEG system. Hence, it is easier to overcome the nonradiative relaxation energy in MEG than that in MYG. The temperature dependence of Eu^3+^ can be expressed by the following equation^43^

(2) $$W_{n}(T)\;=\;W_{n}(0){\left(1\;-\;\exp^{-hv/kT}\right)}^{-n}$$

where *W_n_*(0) is the spontaneous emission probability. *W_n_*(*T*) represents the temperature‐dependent decay rate, which gets smaller with Eu^3+^ substituting Y^3+^. These results indicate that the decreasing nonradiative relaxation generates a lower thermal quenching process. In addition, thermal conductivity is also an important factor to influence the thermal quenching behavior of phosphor.^44^ Generally, high thermal conductivity possesses a faster heat dissipation rate, remarkably helping to decrease thermal quenching loss. In Figure 4b,, both thermal conductivity of MYG:*x*Eu^3+^ (*x* = 0, 0.8, 1) and MYG:*y*Eu^3+^, 0.01Mn^4+^ (*y* = 0, 0.6, 1) display a linear enhancement with increasing Eu^3+^ concentration. This result also provides an evidence for the successful design of antithermal‐quenching properties in current phosphor.

**Figure 5:**
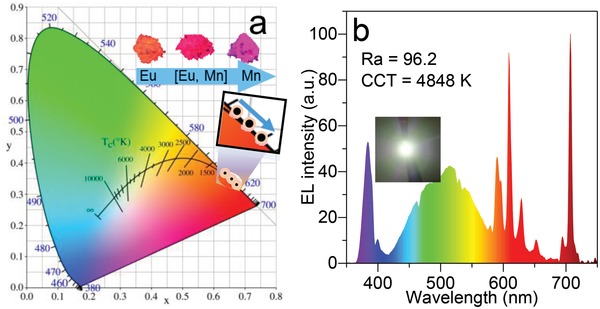
a) CIE chromaticity coordinates and digital photoluminescence photographs (λ_ex_ = 365 nm) of the representative MYG: Eu^3+^, MYG: 0.7Eu^3+^, Mn^4+^, and MYG: Mn^4+^ samples. b) The electroluminescent spectrum of the fabricated w‐LEDs device.

The CIE chromaticity coordinates and photoluminescence photographs (under 365 nm irradiating) for MYG:Eu^3+^ and MYG:*y*Eu^3+^, Mn^4+^ (*y* = 0, 0.7) phosphors exhibit high color purity due to the linearly narrow‐band emission of Eu^3+^ and Mn^4+^ (Figure 5a). MYG:Eu^3+^ presents orangish‐red light at (0.637, 0.362). MYG:Mn^4+^ shows deep‐red light at (0.667, 0.332). The photoluminescence color is tuned to bright red light at (0.652, 0.348) in MYG:0.7Eu^3+^, Mn^4+^. The w‐LEDs device was fabricated by blue BAM: Eu^2+^, green (Ba,Sr)_2_SiO_4_: Eu^2+^, red MYG:0.7Eu^3+^, Mn^4+^ phosphors, and 380 nm InGaN chip (**Figure** **5**b). Under 200 mA current driving, the fabricated w‐LEDs exhibits excellent warm white light with a low corrected color temperature (CCT = 4848 K) and high color rendering index (*R*_a_ = 96.2). In CIE coordinates diagram, the obtained white light locates at (0.322, 0.388). The luminous efficiency reaches 20.1 lm W^−1^, which is lower than commercially Sr[LiAl_3_N_4_]:Eu^2+^ (114 lm W^−1^).^45^ The luminous efficiency can be further improved by preparation optimization. These results demonstrate that red MYG:*y*Eu^3+^, Mn^4+^ (0 ≤ *y* ≤ 1) is a promising candidate for warm w‐LEDs applications.

**Figure 6:**
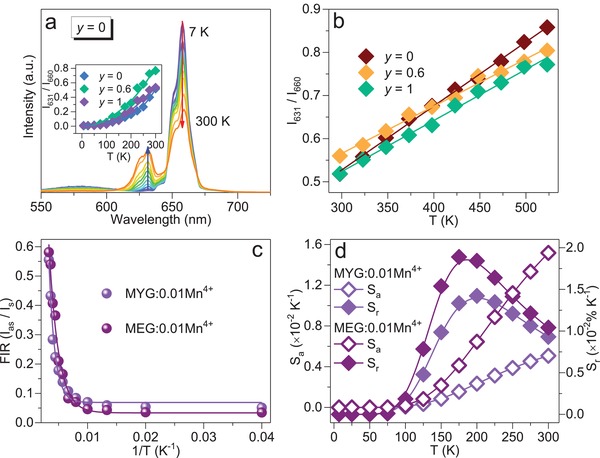
a) Temperature‐dependent PL spectra of MYG:0.01Mn^4+^ from 7–300 K (λ_ex_ = 420 nm), inset plots the integrated intensity ratio *I*_631_/*I*_660_ of 631 and 660 nm. b) Integrated intensity ratio *I*_631_/*I*_660_ as function of temperature (from 298 to 523 K) in MYG:*y*Eu^3+^, 0.01Mn^4+^ (*y* = 0, 0.6, 1) samples. c) Experimental measured and fitted plots of FIR (*I*_as_/*I*_s_) versus temperature (7–300 K) for MYG:0.01Mn^4+^ and MEG:0.01Mn^4+^ samples. d) Absolute sensitivity *S*_a_ and relative sensitivity *S*_r_ versus temperature (7–300 K) in MYG:0.01Mn^4+^ and MEG:0.01Mn^4+^ samples.

**Figure** **6**a displays temperature‐dependent PL spectra of MYG:*y*Eu^3+^, 0.01Mn^4+^ (0 ≤ *y* ≤ 1) in the region of 7–300 K (λ_ex_ = 420 nm). Obviously, the antistokes peak *v*_6_′ of Mn^4+^ in MYG:0.01Mn^4+^ completely disappears at 7 K. When rising temperature, the antistokes peak *v*_6_′ gradually elevates with stokes peak *v*_4_ simultaneously weakening. The integrated intensity ratio of antistokes *v*_6_′ and stokes peak *v*_4_ (*I*_631_/*I*_660_) evidently enhances from 0 (at 7 K) to 0.52, 0.77, 0.54 (at 300 K) for *y* = 0, 0.6, 1. In the region of 298–523 K, *I*_631_/*I*_660_ values also exhibit gradually increasing trend with rising temperature, which even reaches 0.85 at 523 K for MYG:0.01Mn^4+^ (Figure 6b). The above phenomenon is mainly ascribed to the Mn^4+^ unshielded 3d electrons configuration. Previously, it was reported that double perovskite A_2_LaNbO_6_: Mn^4+^, Eu^3+^ (A = Ba, Ca) phosphors can have a potential application in optical temperature sensing field.^46^ To clarify the accurate intensity variation in MYG:*y*Eu^3+^, 0.01Mn^4+^ (0 ≤ *y* ≤ 1), fluorescence intensity ratio (FIR) technique is adopted, which is the self‐referencing optical thermometry based on fluorescence integrated intensity ratio. According to Struck and Fonger theory, the relationship between temperature and integrated PL intensity of the Mn^4+^ antistokes peak *v*_6_′ (*I*_as_) and stokes peak *v*_4_ (*I*_s_) can be expressed as^47^, ^48^

(3) $$\text{FIR}=\frac{I_{\text{as}}}{I_{s}}\approx \text{A}\exp\left(-\Delta E/k_{\text{B}}T\right)+B$$

where A is the proportional parameter, *k*_B_ is the Boltzmann constant, B is an offset parameter and ΔE is the required activation energy to promote the electron from its emitting state to the quenching state. As shown in Figure 6c, for MYG:0.01Mn^4+^ and MEG:0.01Mn^4+^ samples, the measured plots of FIR versus temperature can be fitted well with equation (3). The absolute and relative temperature sensitivity (donated as *S*_a_, *S*_r_) could be further described as the following equations^49^, ^50^, ^51^, ^52^

(4) $$S_{a}\;=\;\frac{d\text{FIR}}{d\text{T}}\;=\;\text{C}\exp(-\Delta E\text{/}k_{\text{B}}T)\;\times \;\frac{\Delta E}{k_{B}T^{2}}$$

(5) $$\begin{array}{c} S_{r}=100\%\times \left|\frac{1}{\text{FIR}}\frac{d\text{FIR}}{dT}\right|\\ =100\%\times \frac{\text{C}\exp(-\Delta E/k_{\text{B}}T)}{\text{D}+\text{C}\exp(-\Delta E\text{/}k_{\text{B}}T)}\times \frac{\Delta E}{k_{\text{B}}T^{2}} \end{array}$$

where C and D are the related constants. As displayed in Figure 6d, for MEG:0.01Mn^4+^ sample, the maximum value of *S*_a_ and *S*_r_ are 0.015 K^−1^ at 175 K, 0.019% K^−1^ at 300 K, which is bigger than that of MYG:0.01Mn^4+^ sample (*S*_a_ = 0.005 K^−1^ at 300 K, *S*_r_ = 0.014% K^−1^ at 200 K). MEG:Mn^4+^ phosphor is more suitable for optical thermometry sensors materials. In addition, as seen in Table S3 in the Supporting Information, the as‐prepared MEG:0.01Mn^4+^ sample even presents a much higher sensitivity than the previously reported Mn^4+^ doped temperature sensing phosphors.^48^, ^53^, ^54^, ^55^, ^56^, ^57^ This result indicates that as‐prepared MEG:0.01Mn^4+^ sample could also be a promising candidate in the application of low‐temperature thermometry sensors.

## 3 Conclusion

In summary, we propose a novel strategy to develop antithermal‐quenching red phosphor with consecutive emission enhancement (120%) from 298 to 523 K. This behavior is mainly attributed to thermally stable Mn^4+^ stokes peaks *v*_4_ (P_473 K_/*P*_0_ exceeds 90%) and antithermal‐quenching antistokes peak *v*_6_′ (P_473 K_/*P*_0_ ˃ 125%) by designing energy transfer from Eu^3+^ to Mn^4+^. The photoluminescence color is tuned from orangish‐red to deep‐red light by just changing Eu^3+^ concentration, minimizing the efficacy loss at long wavelengths. The internal quantum yield is obviously enhanced from 15.1% to 76.5%. The fabricated w‐LEDs with MYG:0.7Eu^3+^, Mn^4+^ as red component exhibits warm white light of CCT = 4848 K and *R*_a_ = 96.2. MEG:0.01Mn^4+^ has an advantage over other Mn^4+^‐doped phosphors in optical thermometry sensors (*S*_a_ = 0.015 K^−1^ at 175 K and *S*_r_ = 0.019% K^−1^ at 300 K). The concept of designing Eu^3+^ → Mn^4+^ energy transfer provides a novel strategy to explore antithermal‐quenching and color‐adjustable red phosphors for various optical applications.

## 4 Experimental Section

##### Materials and Preparation

Mg_3_Y_2(1−_ *_x_*_)_Ge_3_O_12_:*x*Eu^3+^ (0 ≤ *x* ≤ 1) (abbreviated as MYG:*x*Eu^3+^) and Mg_3_Y_2(1−_ *_y_*_)_Ge_3_O_12_:*y*Eu^3+^, 0.01Mn^4+^ (0 ≤ *y* ≤ 1) (abbreviated as MYG:*y*Eu^3+^, 0.01Mn^4+^) samples were prepared via a Pechini sol–gel route. Mg(NO_3_)_2_∙6H_2_O (AR), Y_2_O_3_ (≥ 99.99%), Eu_2_O_3_ (≥ 99.99%), GeO_2_ (99.999%), MnCl_2_ ∙ 4H_2_O (AR), Li_2_CO_3_ (99.99%), HNO_3_ (65–68%), NH_4_OH (25–28%), citric acid and polyethylene glycol (PEG, molecular weight = 20 000) were raw materials. All the chemicals were directly used without further purification. The concentration of Mn^4+^ was 1 mol% of Mg^2+^, additional 1.5 mol% Li^+^ was added as charge compensation. First, stoichiometric amounts Y_2_O_3_, Eu_2_O_3_ were dissolved in nitric acid with stirring and heating, after the mixed solution turned to be transparent, Mg(NO_3_)_2_ ∙ 6H_2_O, MnCl_2_∙4H_2_O, and Li_2_CO_3_ were then added, forming solution A. GeO_2_ was dissolved in ammonium hydroxide with stirring and heating, forming solution B. Second, when solution A and B cooled naturally to room temperature, solution B was slowly decanted into solution A. Thirdly, citric acid (*n*_citric acid_:*n*_metal ions_ = 2:1) and polyethylene glycol were successively added into the resultant solution with fully stirring for 1 h. The homogenous gels were formed after heating in a water bath at 75 °C for 24 h. Finally, the dried gels were preheated at 500 °C for 5 h in air, and the precursors were grounded and heated at 1450 °C for 5 h in air. When the furnace slowly cooled down to room temperature, the sintered powders were ground again, yielding the final phosphor powders.

##### w‐LED Fabrication

The w‐LEDs device was fabricated by using a n‐UV InGaN chip with the mixed RGB phosphors of blue BAM:Eu^2+^, green (Ba,Sr)_2_SiO_4_: Eu^2+^, and red MYG: 0.7Eu^3+^, 0.01Mn^4+^ phosphors. At first, quantified RGB phosphors were weighted and evenly mixed with silicone resins A and B (A:B = 1:1) in the agate mortar. Then, the mixture was coated on a 380 nm InGaN chip. The packaged devices were cured in an oven at 120 °C for 12 h to form the final w‐LEDs devices.

##### Characterization

The structure and phase purity were performed on a D8 Focus diffractometer at a scanning rate of 1° · min^−1^ in the 2θ range from 5° to 120° with Ni‐filtered Cu‐Kα (λ = 1.540598 Å). XRD Rietveld profile refinements of the structural models and texture analysis were measured with the use of General Structure Analysis System (GSAS) software. The morphology, EDS, and elemental mapping analysis of the samples were inspected by using a field‐emission scanning electron microscope (FE‐SEM, S‐4800, Hitachi, Tokyo, Japan). The photoluminescence excitation (PLE) and emission (PL) spectra were measured by fluorescence spectrometer (Fluoromax‐4P, Horiba Jobin Yvon, New Jersey, USA) equipped with a 150 W xenon lamp as the excitation source, and both excitation and emission spectra were set up to be 1.0 nm with the width of the monochromator slits adjusted as 0.50 nm. The diffuse reflectance spectra were measured by UV–visible diffuse reflectance spectroscopy (UV‐2550PC, Shimadzu Corporation, Kyoto, Japan). The photoluminescence quantum yield (QY) was measured by an absolute PL quantum yield measurement system (C9920‐02, Hamamatsu Photonics K.K., Japan). The photoluminescence decay curves and time‐resolved photoluminescence spectra were obtained from a Lecroy Wave Runner 6100 Digital Oscilloscope (1 GHz) using a tunable laser (pulse width = 4 ns, gate = 50 ns) as the excitation (Continuum Sunlite OPO). Raman spectra were obtained on Raman spectrometer (JYT6400) with using a 512 nm laser. The thermal conductivities of samples were performed by using a thermal conductometer (XIATECH TC30000E) with a heat sensor (9P3000E). All the above measurements were performed at room temperature. Temperature‐dependent PL spectra (10–300 K and 298–573 K) were recorded on a fluorescence spectrophotometer (Edinburgh Instruments FLSP‐920) with a temperature controller. The temperature‐dependent photoluminescence decay curves were obtained from a Lecroy Wave Runner 6100 Digital Oscilloscope (1 GHz) with a temperature controller.

## Conflict of Interest

The authors declare no conflict of interest.

## Supporting information

Supporting Information
