# Supplementary material for: Strategies for Designing Antithermal‐Quenching Red Phosphors
Source: Adv Sci (Weinh). 2020 Feb 29;7(8):1903060. doi: 10.1002/advs.201903060 (PMC7175321; doi:10.1002/advs.201903060)
Supplement: Supplementary file 1 — Supporting Information [file ADVS-7-1903060-s001.pdf]

Copyright WILEY-VCH Verlag GmbH & Co. KGaA, 69469 Weinheim, Germany, 2020.

ADVANCED SCIENCE
Open Access

Supporting Information

for *Adv. Sci.,* DOI: 10.1002/advs.201903060

Strategies for Designing Antithermal-Quenching Red Phosphors

Yi Wei, Hang Yang, Zhiyu Gao, Yixin Liu, Gongcheng Xing, Peipei Dang, Abdulaziz A. Al Kheraif, Guogang Li, Jun Lin,* and Ru-Shi Liu**

Copyright WILEY-VCH Verlag GmbH & Co. KGaA, 69469 Weinheim, Germany, 2020.

Supporting Information

Strategies for Designing Anti-thermal-quenching Red Phosphors

Yi Wei, Hang Yang, Zhiyu Gao, Yixin Liu, Gongcheng Xing, Peipei Dang, Abdulaziz A. Al Kheraif , Guogang Li, * *Jun Lin,* * *and Ru-Shi Liu* *

Results and Discussion

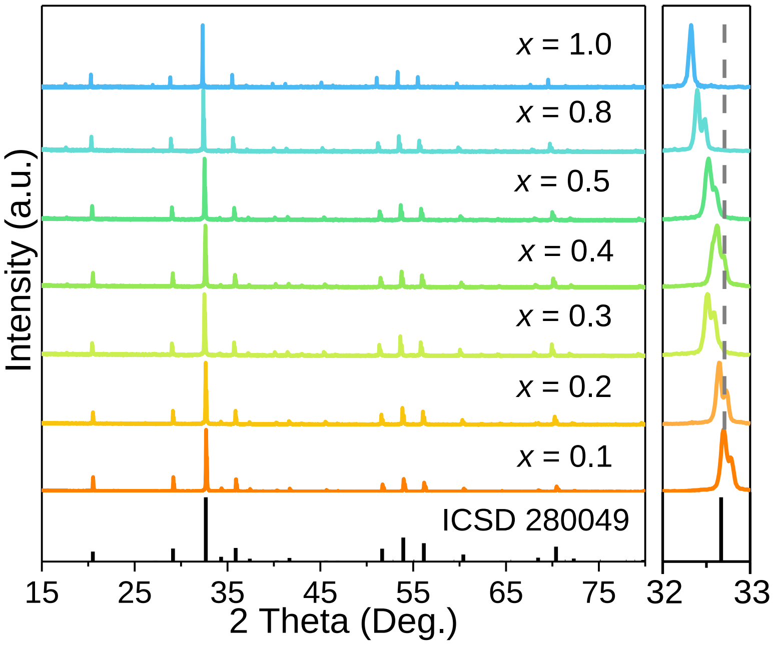

Figure S1. XRD patterns of MYG:*x*Eu^{3+} ($0 \leq x \leq 1$), the standard card refers as $Mg_3Y_2Ge_3O_{12}$ (ICSD. 280049).

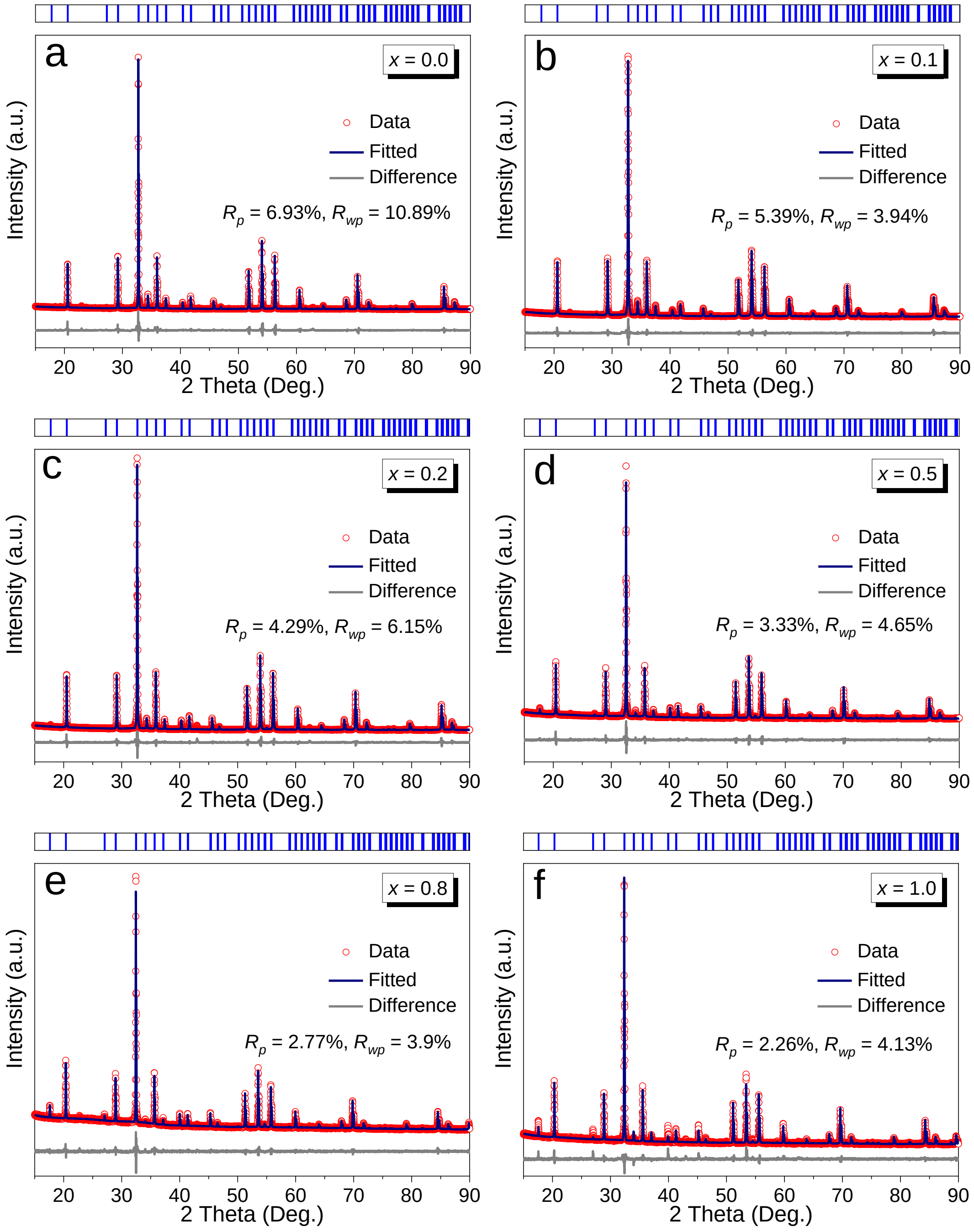

Figure S2. The Rietveld refinement for MYG:xEu^{3+} ($0 \leq x \leq 1$) samples, (a) x = 0.0; (b) x = 0.1; (c) x = 0.2; (d) x = 0.5; (e) x = 0.8 and (f) x = 1.0, where red, navy, grey, blue lines stand for the measured data, fitted data, the difference between measured and fitted data, and bragg position, respectively.

Table S1. Lattice structure, lattice parameters and refinement factors for MYG:xEu^{3+} ($0 \leq x \leq 1$) and MYG:xEu^{3+},.0.01Mn^{4+} ($0 \leq y \leq 1$)samples based on the Rietveld refinement XRD patterns.

Samples	Space group	Lattice parameters		*R*-factors		
		a (Å)	V (Å^3)	R_p (%)	R_{wp} (%)	χ^2
$x = 0$		12.23157(4)	1829.982(11)	6.93	10.89	11.74
$x = 0.1$		12.25701(6)	1841.421(14)	5.39	3.94	3.27
$x = 0.2$		12.23043(7)	1829.469(17)	4.29	6.15	4.547
$x = 0.5$		12.26768(6)	1846.235(15)	3.33	4.65	2.856
$x = 0.8$		12.30115(5)	1861.391(14)	2.77	3.9	1.961
$x = 1$	Cubic phase	12.33906(7)	1878.653(18)	2.26	4.13	2.96
$y = 0$	*Ia-3d*	12.23154(5)	1829.969(13)	6.52	10.3	10.16
$y = 0.1$		12.29048(5)	1856.548(12)	5.4	8.69	8.28
$y = 0.3$		12.27065(5)	1847.577(13)	4.02	5.97	4.231
$y = 0.5$		12.29923(5)	1860.518(14)	3.51	5.31	3.215
$y = 0.8$		12.33451(5)	1876.576(14)	2.92	4.71	3.200
$y = 1.0$		12.37118(5)	1893.362(13)	2.87	4.06	2.481

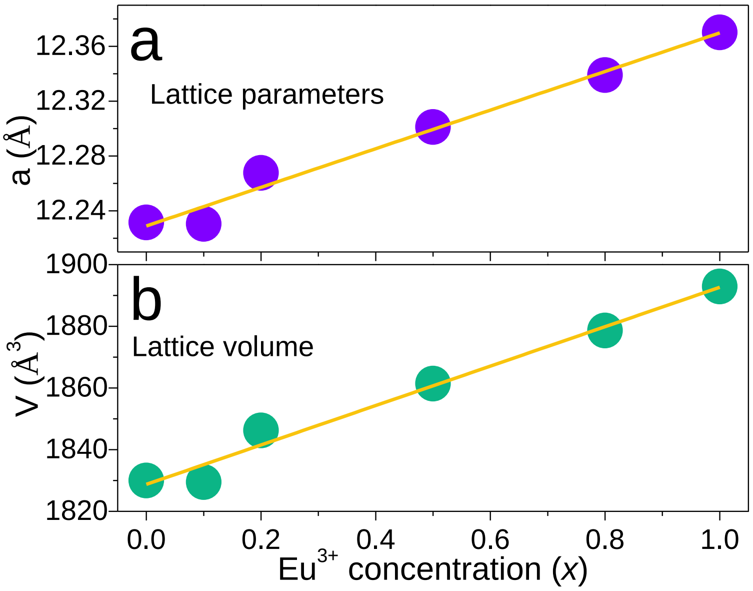

Figure S3 (a, b) The lattice parameters a and cell volume V as a function of Eu^{3+} concentration in MYG:xEu^{3+} ($0 \leq x \leq 1$).

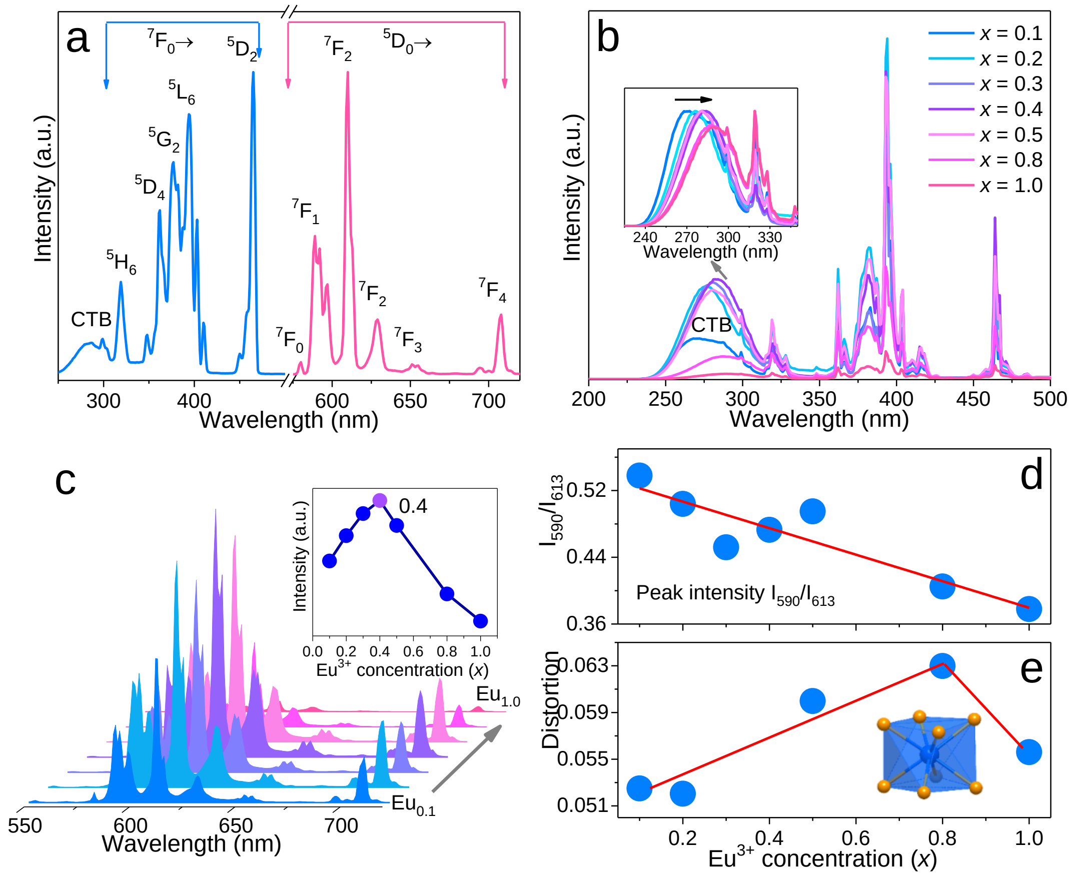

Figure S4 (a) The schematic electron transition of the PLE and PL spectra in MYG:0.4Eu^{3+} samples at room temperature. (λ_{ex} = 393 nm, and λ_{em} = 610 nm) (b)The PLE spectra of MYG:*x*Eu^{3+} ($0.1 \leq x \leq 1$) (λ_{em} = 610 nm). (c) The PL spectra of MYG:*x*Eu^{3+} ($0.1 \leq x \leq 1$) samples (λ_{ex} = 393 nm), the inset is the integrated intensity as function of Eu^{3+} concentration in MYG:*x*Eu^{3+} ($0 \leq x \leq 1$). (d) The peak intensity ratio at 590 nm and 613 nm (I_{590}/I_{613}) and (e) lattice distortion degree for Y/Mg1/EuO_8 polyhedron *vs* Eu^{3+} concentration.

As increasing Eu^{3+} substitution concentration, the profile and shape of excitation spectra keep nearly unchanged, the intensities appear a tendency of first increase and subsequent decrease. The optimal concentrations for CTB, excitation peak at 394 nm and 464 nm are 0.4, 0.2, and 0.4 respectively. It is noteworthy that a CTB redshift appears with increasing Eu^{3+} concentration, owing to the replacement for larger Eu^{3+} ions. Generally, the larger are metal ions, the weaker is M^{3+}-O^{2-} covalency. As a consequence, when Eu^{3+} ions replace smaller Y^{3+} ions, the covalency of M^{3+}-O^{2-} gradually decreases, the sideband of CTB slightly shift to the long-wavelength direction. Upon 394 nm exciting, photoluminescence emission (PL) spectra of MYG:*x*Eu^{3+} ($0.1 \leq x \leq 1$) demonstrate the typical Eu^{3+} luminescence with the peak at around 610 nm owing to $^5D_0 \rightarrow ^7F_2$ electron transition (Figure S4a, S4c). As Eu^{3+} concentration increases, PL intensity first enhances and then decreases. Simultaneously, the integrated PL intensity of various Eu^{3+} ions plots that the optimal concentration is x = 0.4. When the Eu^{3+} content exceeds 0.4, nonradiative energy transition causes concentration quenching. To validate the intrinsic mechanism of concentration, we calculate the critical distance (R_c) of the interaction between Eu^{3+} by using the following formula:

$$R_c = 2\left[\frac{3V}{4\pi x_c Z}\right]^{1/3} \qquad (1)$$

where x_c represents the critical concentration, Z stands for the number of formula units per unit cell, V is the lattice volume. The calculated R_c equals to 10.3 Å, which is longer than the nearest distance between Y^{3+} ions (3.745 Å, obtained by Rietveld refinement), indicating that the

concentration quenching is mainly caused by the energy transfer from one Eu^{3+} ion to another. Since R_c value is larger than 5 Å, the main reason for energy is multipolar interactions. It is noteworthy mentioning that MEG sample still displays obvious red emission, illustrating that the Eu^{3+} ions could act as host cation as well as an activator.

The distortion index (D) of [Y/Eu/MgO_8] dodecahedron could be analyzed with the following equation [1-2]:

$$D = \frac{1}{n}\sum_{i=1}^{n}\frac{|d_i - d_{av}|}{d_{av}} \qquad (2)$$

where d_i is the distance from Ba to the ith coordinating O atoms, d_{av} is the average Ba−O bond length, and n is the coordinate numbers. Obviously, D values of MYG:$x$$Eu^{3+}$ show a trend of first rising and subsequent falling, reaching a maximum value at x = 0.8 (Figure S4e). At $0 < x < 1$, the dodecahedron is co-occupied by larger Y^{3+}, Eu^{3+} ions and smaller Mg^{2+} ions (CN = 8, r = 0.89 Å), and thus the [Y/Eu/MgO_8] polyhedron is seriously distorted. As the radii difference between Eu^{3+} and Mg^{2+} is larger than that between Y^{3+} and Mg^{2+}, the distortion index of [Eu/MgO_8] dodecahedron displays more distortion than [Y/MgO_8].

Table S2. The Atom% of MYG:0.6Eu^{3+}, 0.01Mn^{4+} via EDS measurement

Points	Mg (%)	Y (%)	Eu (%)	Ge (%)	Mn (%)	O (%)
1	20.89	5.45	6.07	16.58	0.19	50.82
2	17.23	4.97	11.49	16.46	0.39	49.46
3	21.8	4.57	5.37	12.54	0.19	55.53
4	19.04	4.1	4.57	14.34	0.12	57.82
5	20.07	4.02	4.13	12.25	0.14	59.39
6	22.51	5.18	9.72	16.93	0.49	45.18
7	18.67	5.37	7.27	16.08	0.37	52.24
8	18.37	5.03	5.7	16.58	0.17	54.15
9	22.82	5.03	5.58	14.14	0.13	52.31
10	18.7	5.01	5.32	15.85	0.15	54.97
11	18.99	4.96	8.29	20.27	0.24	48.25
12	20.05	5.44	6.16	14.02	0.14	54.18
13	21.21	4.86	5.12	11.66	0	57.15
14	20.41	5.51	6.16	14.16	0.28	53.49
15	19.94	5.3	7.21	14.96	0.4	51.19
Average	20.05	4.99	6.544	15.12	0.23	53.08
Atom ratio	3.98	0.99	1.30	3.00	0.05	10.53

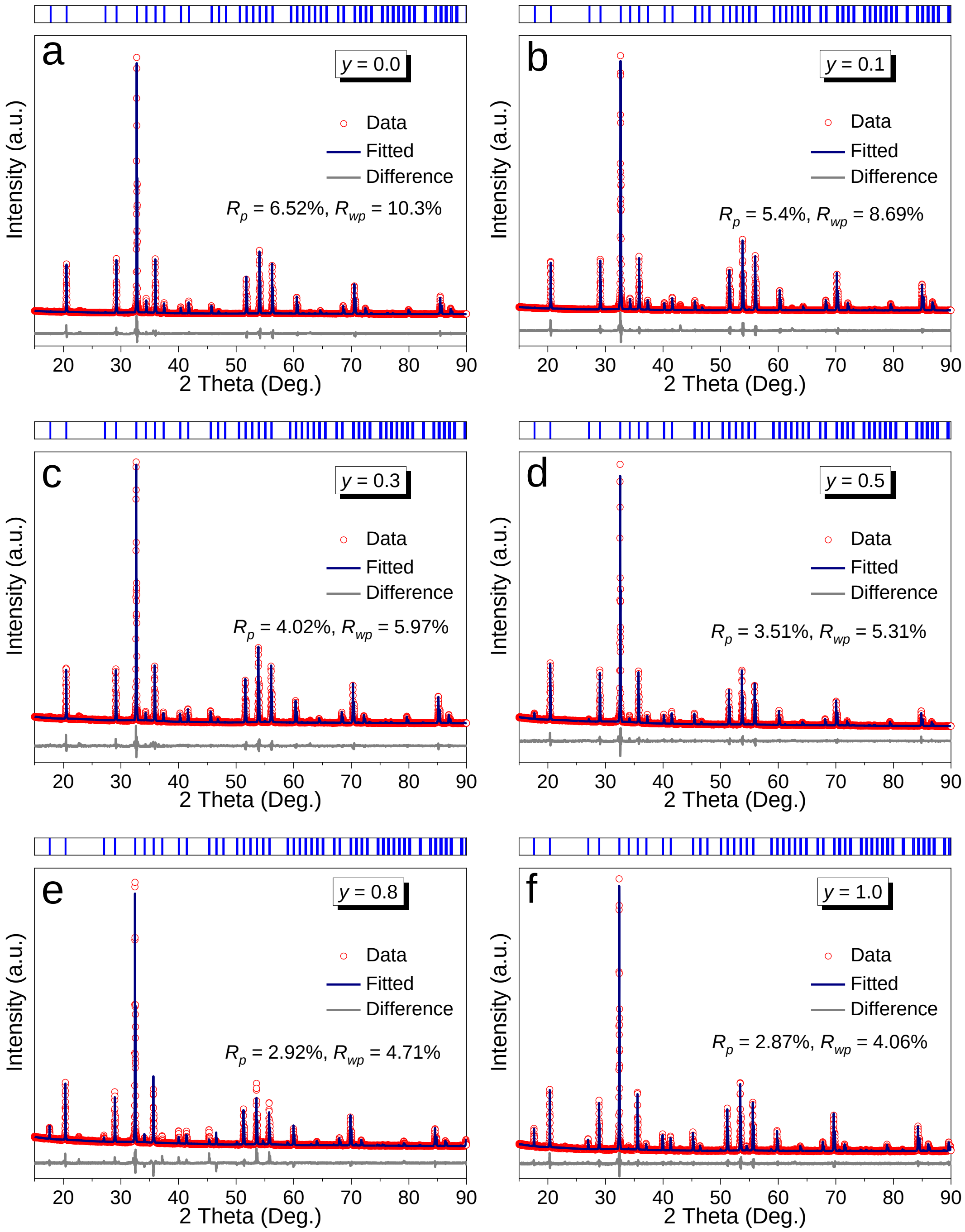

Figure S5. The Rietveld refinement for MYG:yEu^{3+}, 0.01Mn^{4+} ($0 \leq y \leq 1$) samples, (a) $y = 0$; (b) $y = 0.1$; (c) $y = 0.3$; (d) $y = 0.5$; (e) $y = 0.8$; (f) $y = 1.0$, where red, navy, grey, blue lines stand for the measured data, fitted data, the difference between measured and fitted data, and bragg position, respectively.

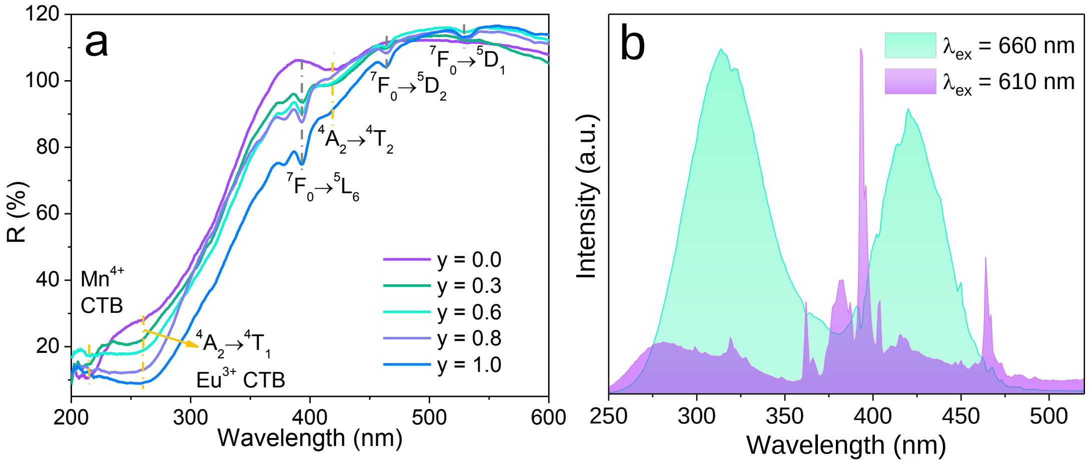

Figure S6. (a) The DR spectra of MYG:*y*Eu^{3+}, $0.01Mn^{4+}$ ($0 \leq y \leq 1$) samples. (b) PLE spectra of MYG:$0.5Eu^{3+}$, $0.01Mn^{4+}$ sample (λ_{em} = 610 nm and λ_{em} = 660 nm).

From the DR spectra in Figure S6a, it is noted that MYG:$0.01Mn^{4+}$ sample exhibits two broad bands from 200 nm to 460 nm. The strong one in the region of 200-350 nm centered at 260 nm originates from the $^4A_2 \rightarrow ^4T_1$ transition of Mn^{4+}, while the weak one in the region of 390-460 nm centered at 420 nm is ascribed to $^4A_2 \rightarrow ^4T_2$ transition of Mn^{4+}. Between the above two broad bands, there is a concave area from 350 nm to 390 nm, and the excitation intensity is weak. As Eu^{3+} ions entering into MYG matrix, the excitation band contains characteristic Mn^{4+} absorption as well as typical Eu^{3+} absorption bands. Therefore, excitation intensity of MYG:Eu^{3+}, Mn^{4+} in n-UV and the blue area gradually enhance with increasing Eu^{3+} content, demonstrating that codoping Eu^{3+} is an effective way to improve Mn^{4+} excitation intensity in the n-UV region.

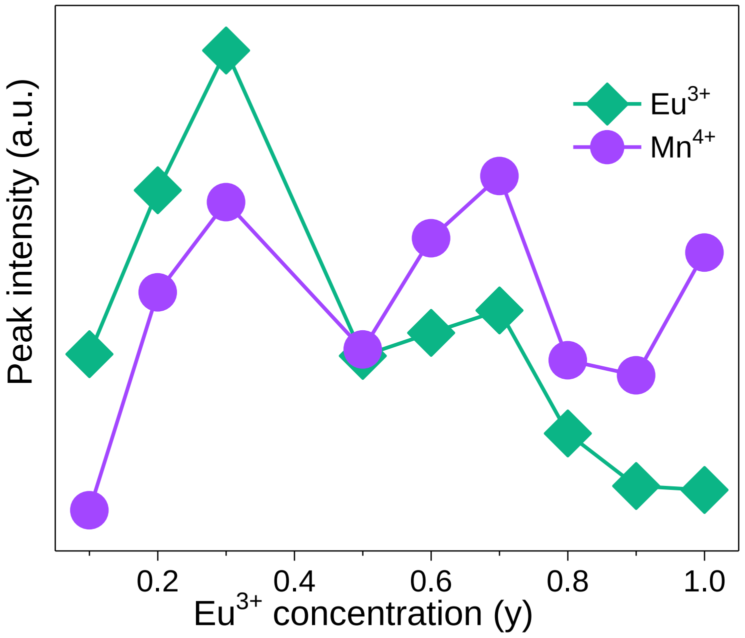

Figure S7. Peak intensity of Eu^{3+} and Mn^{4+} *vs* Eu^{3+} concentration (*y*) in MYG:*y*Eu^{3+}, 0.01Mn^{4+} ($0.1 \leq y \leq 1$) (λ_{ex} = 380 nm).

The relative peak intensities of Eu^{3+} $^5D_0 \rightarrow ^7F_1$ peak and Mn^{4+} $^2E_g \rightarrow ^4A_2$ peak are shown in Figure S7. At $0.1 \leq y \leq 0.5$, the peak intensity of Eu^{3+} is stronger than Mn^{4+}, until $y = 0.5$, the peak intensity of Eu^{3+} almost equals to Mn^{4+}. While *y* exceeds 0.5, Eu^{3+} luminescence is weaker than Mn^{4+}. The main reason is the structural evolution from MYG to MEG matrix due to the slightly different electron configuration. MYG matrix dominates the crystal structure at $y < 0.5$, in which Eu^{3+} mainly acts as an activator. When $y > 0.5$, Eu^{3+} mainly contributes to the matrix framework, forming a MEG matrix, so the luminescence quenching is more serious in MYG.

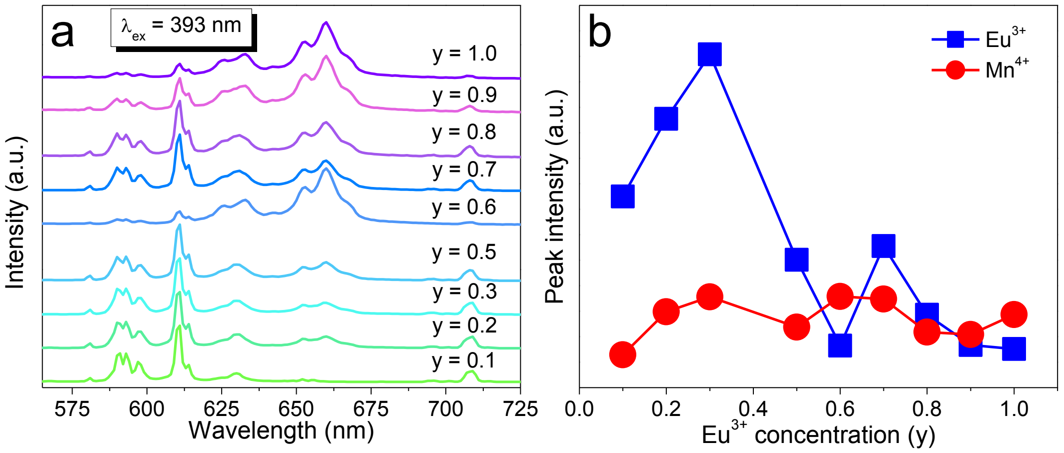

Figure S8. (a) The PL spectra of MYG:yEu^{3+}, 0.01Mn^{4+} ($0.1 \leq y \leq 1$) samples. (b) The relative peak intensity of Eu^{3+} and Mn^{4+} luminescence as function of Eu^{3+} ions concentration (y) in MYG:yEu^{3+}, 0.01Mn^{4+} ($0.1 \leq y \leq 1$).

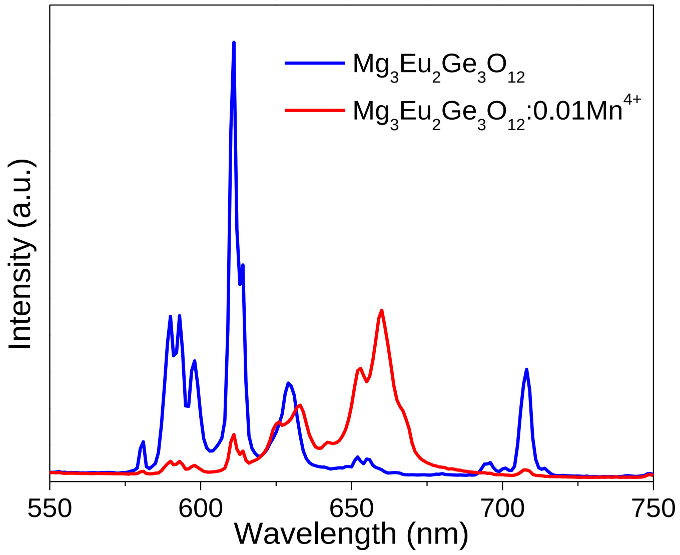

Figure S9. The PL spectra of $Mg_3Eu_2Ge_3O_{12}$ and $Mg_3Eu_2Ge_3O_{12}$:0.01Mn^{4+} samples monitored at 393 nm.

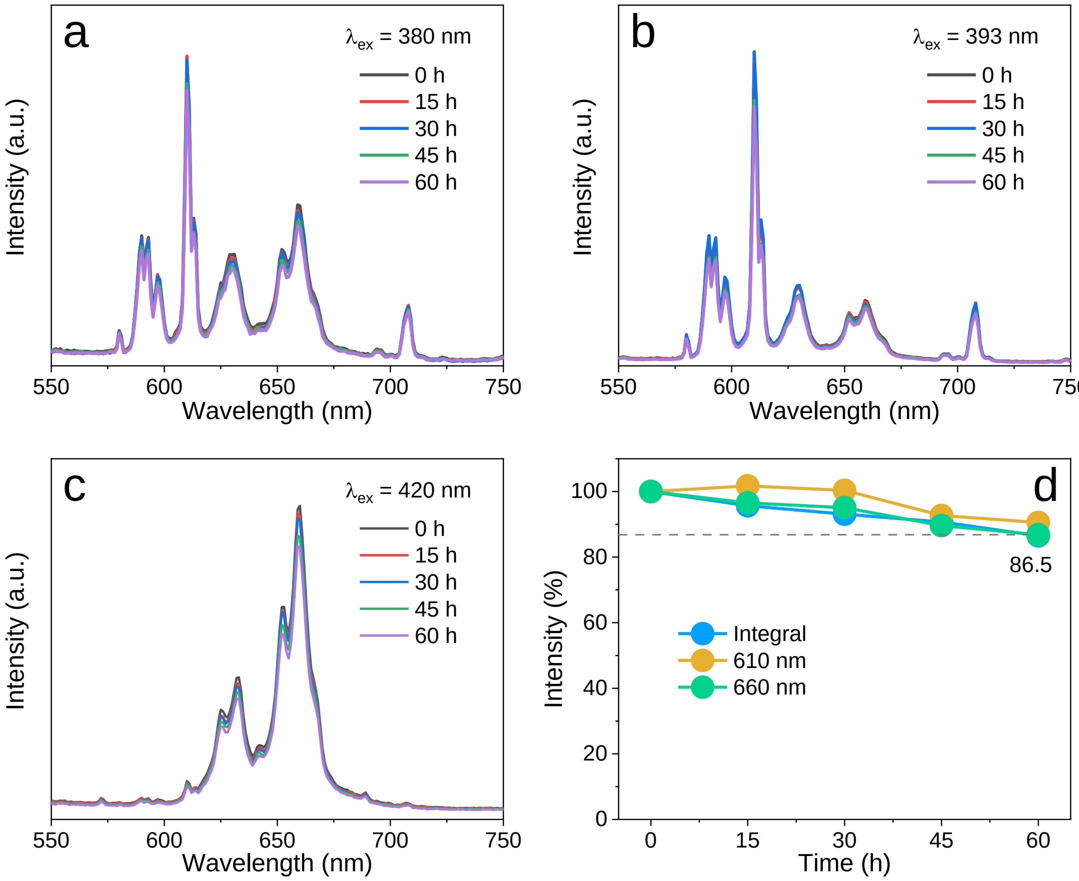

Figure S10. The PL spectra of MYG:yEu^{3+}, 0.01Mn^{4+} ($0.1 \leq y \leq 1$) samples with different time in air (a) λ_{ex} = 380 nm, (b) λ_{ex} = 393 nm and (c) λ_{ex} = 420 nm. (d) The relationship between integrated intensity and time for MYG:0.6Eu^{3+}, 0.01Mn^{4+} sample under 380 nm excitation.

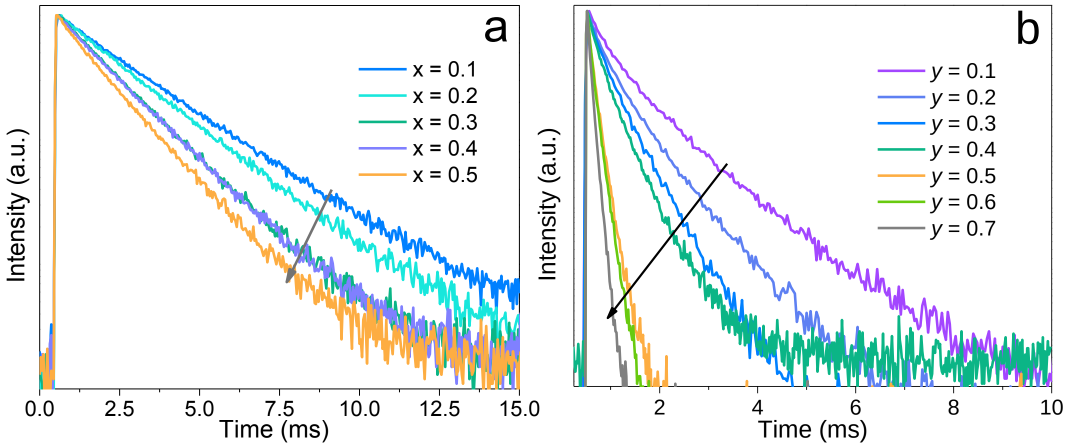

Figure S11. (a) The PL decay curves for MYG:xEu^{3+} ($0.1 \leq x \leq 0.5$) samples monitored λ_{ex} = 393 nm, and λ_{em} = 610 nm. (b)The luminescence decay curves for MYG:yEu^{3+}, 0.01Mn^{4+} ($0.1 \leq y \leq 0.7$) samples monitored λ_{ex} = 393 nm, and λ_{em} = 610 nm.

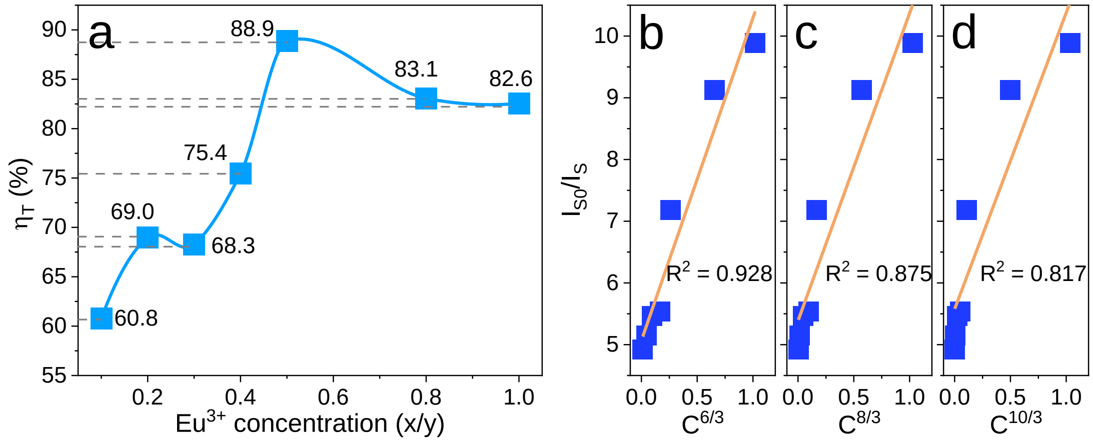

Figure S12. (a) The energy transfer efficiency (η_T) from Eu^{3+} to Mn^{4+} based on the equation of $\eta_T = 1 - \frac{\tau_S}{\tau_{S0}}$, where τ_S and τ_{S0} represent the lifetime value with and without the Mn^{4+} ions, respectively. The relationship of I_{S0}/I_S for Eu^{3+} ions on (b) $C^{6/3}$;(c) $C^{8/3}$; and (d) $C^{10/3}$ for MYG:$y$$Eu^{3+}$, 0.01$Mn^{4+}$ ($0.1 \leq y \leq 1$) samples.

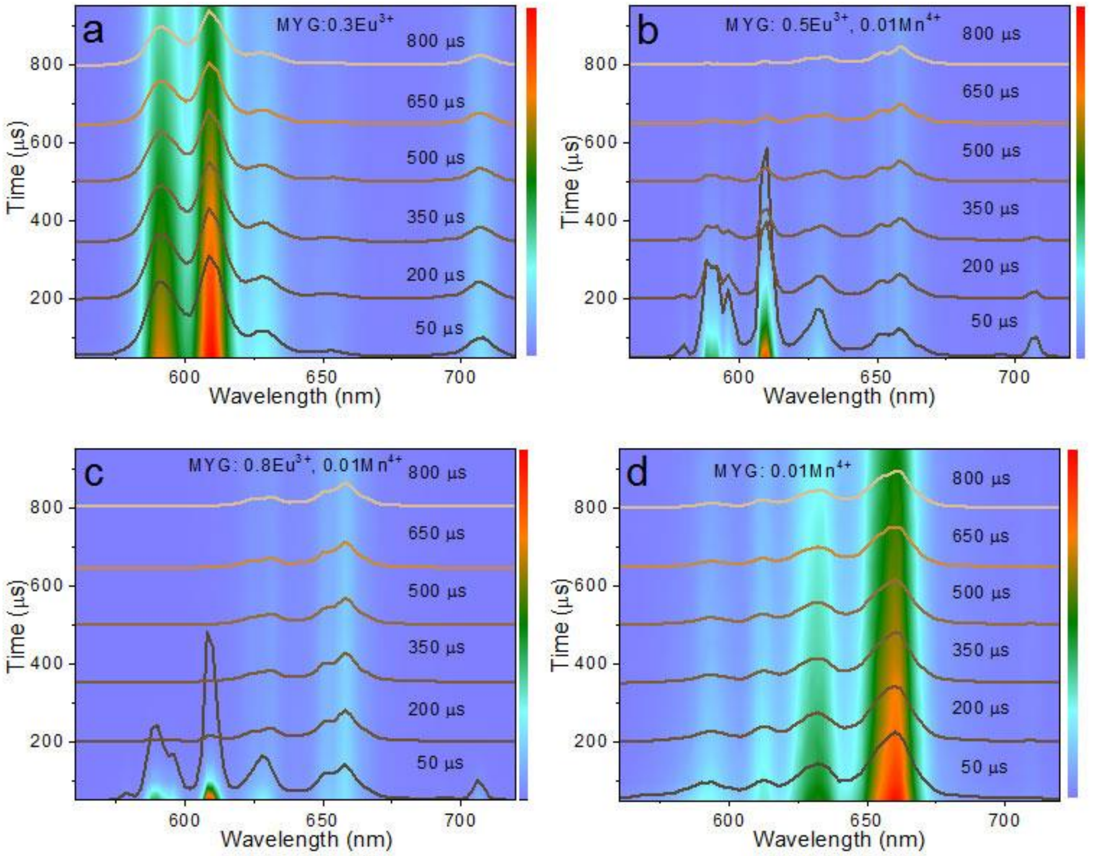

Figure S13. The time-resolved PL spectra in the time range of 50-950 µs and the representative time decay curvy at time 50 µs, 200 µs, 350 µs, 500 µs, 650 µs, 800 µs for (a) MYG:0.3Eu^{3+}, (b) MYG:0.5Eu^{3+}, 0.01Mn^{4+}, (c) MYG:0.8Eu^{3+}, 0.01Mn^{4+} and (d) MYG: 0.01Mn^{4+} samples.

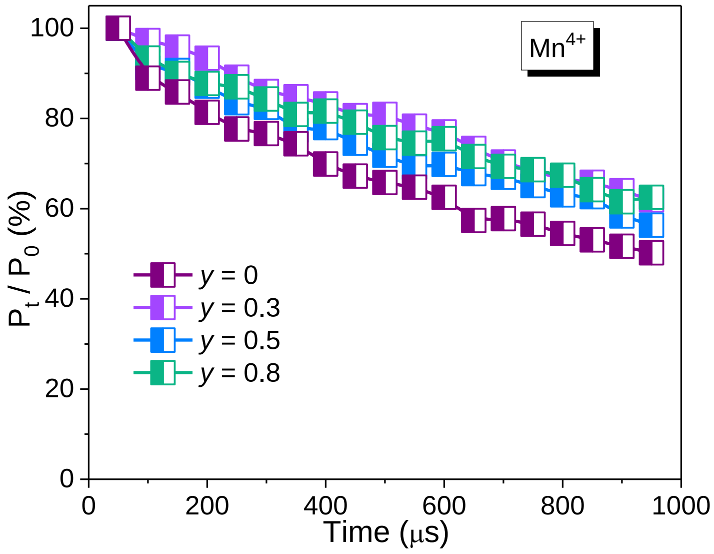

Figure S14. The peak intensity ratio (P_t/P_0, P_t represents peak intensity at time t, P_0 represents peak intensity at 50 μs) as functions of irradiation time in MYG:*y*Eu^{3+}, 0.01Mn^{4+} (*y* = 0, 0.3, 0.5, 0.8).

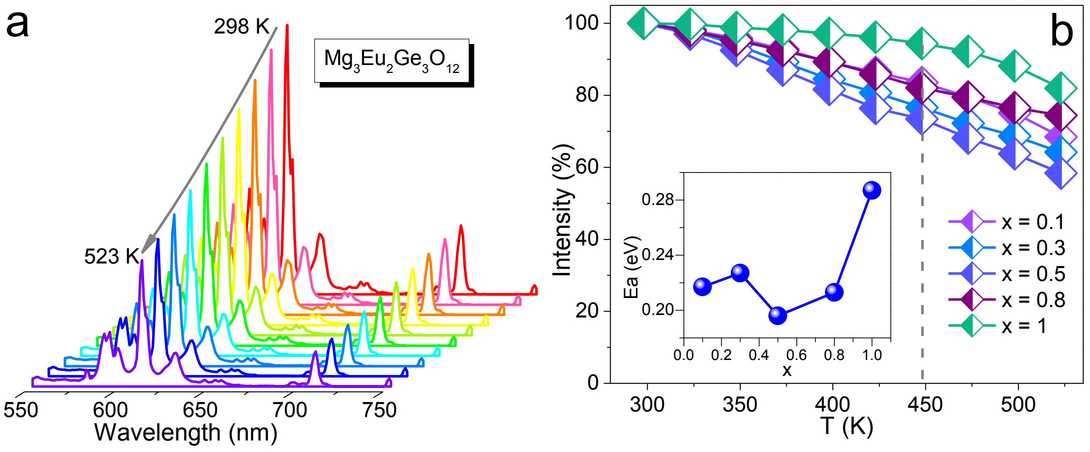

Figure S15. (a) The temperature dependent PL spectra of $Mg_3Eu_2Ge_3O_{12}$ from 298 K to 523 K with 25 K an interval. (b) The relative integrated intensity *vs* heating temperature in MYG:$x$$Eu^{3+}$ ($0.1 \leq x \leq 1$) monitored at λ_{ex} = 393 nm, the inset is the active energy (E_a) as a function of Eu^{3+} concentration (x).

To the best of our knowledge, TQ is usually in negative correlation with activation energy E_a, which could be calculated by using the Arrhenius equation:

$$I_T = \frac{I_0}{1 + c\exp(-\frac{E_a}{kT})} \qquad (3)$$

Rearranging this equation into

$$\ln(I_0 / I_T - 1) = -\frac{E_a}{kT} \qquad (4)$$

where c is a constant, k is Boltzmann's constant with a value of 8.62×10^{-5} eV·K^{-1}, I_0 is the initial emission intensity measured at RT, I_T represents the emission intensity measured at different temperatures, and E_a is the activation energy for the TQ. According to equation (5) and (6), E_a can be obtained by plotting the $\ln(I_0/I_T-1)$ *vs* $1/kT$ curve. The calculated E_a value is displayed in the inset of Figure S13b, as Eu^{3+} concentration increase from 0.1 to 1, the E_a value gradually promotes from 0.217 eV to 0.287 eV, which is the main reason for the declining TQ.

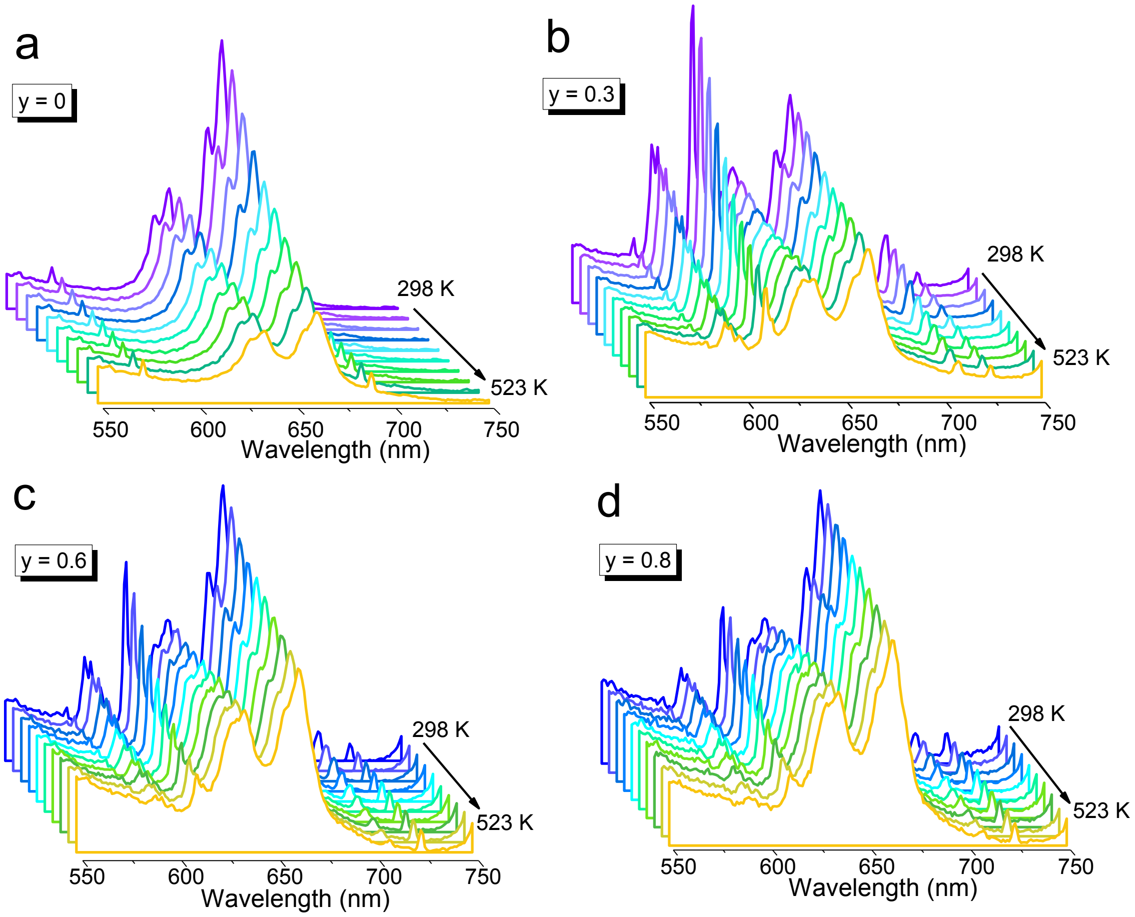

Figure S16. The temperature-dependent PL spectra of MYG:$y$$Eu^{3+}$, 0.01$Mn^{4+}$ (y = 0, 0.3, 0.6 and 0.8) from 298 K-523 K, monitored at λ_{ex} = 380 nm.

At y = 0, the peaks at 633 nm and 660 nm of Mn^{4+} gradually decrease with increasing temperature. The relative peak intensity ratio P_T/P_0 (P_T represents peak intensity at temperature T, P_0 represents peak intensity at 298 K) of T = 473 K could remain 78% and 51% for the peaks at 633 nm and 660 nm, respectively. After doping Eu^{3+} into the matrix, at y = 0.6, there exist three emission peaks at 610 nm, 633 nm, 660 nm, which belong to Eu^{3+} $^5D_0$-$^7F_2$, Mn^{4+} anti-stokes v_6' and Mn^{4+} stokes peaks v_4, respectively. When the temperature gradually increases, the $^5D_0$-$^7F_2$ peak and Mn^{4+} stokes peak v_4 simultaneously decrease, and the former ($P_{473\ K}/P_0$ = 52%) shows more seriously descending tendency than the latter ($P_{473\ K}/P_0$ = 74%). Interestingly, the Mn^{4+} anti-stokes peak v_6' shows almost zero quenching ($P_{473\ K}/P_0$ = 99%). When Y^{3+} ions are totally replaced by Eu^{3+} ions to form MEG matrix, Mn^{4+} stokes peak v_4 slightly decreases, whose peak intensity at 473 K keep 90% of the initial intensity at 298 K. Unexpectedly, the Mn^{4+} anti-stokes peak v_6' show a gradual increase tendency where the luminescence intensity at 473 K even reach 125% of the initial intensity at 298 K. Detailed peaks intensities variation of Mn^{4+} stokes peak v_4 and anti-stokes peak v_6' in MYG:$y$$Eu^{3+}$, 0.01$Mn^{4+}$ ($0 \le y \le 1$) are summarized in Figure 5a. Obviously, both PL intensities at stokes peak v_4 (660 nm) and anti-stokes peak v_6' (633 nm) are dramatically enhanced with increasing Eu^{3+} content, where the $P_{473\ K}/P_0$ of stokes peak v_4 increase from 51% to 90%, and the $P_{473\ K}/P_0$ of anti-stokes peak v_6' increase from 78% to 125%. At high Eu^{3+} concentration (y = 0.8, 1), little TQ with less than 10% emission loss appears in Mn^{4+} stokes peaks v_4, while anti-stokes peak v_6' presents extraordinarily anti-thermal-quenching properties. The above results indicate that the abnormally anti-thermal-quenching appearance is mainly ascribed to the thermally stable Mn^{4+} stokes peaks v_4 and anti-thermal-quenching anti-stokes peak v_6' based on Eu^{3+}→Mn^{4+} energy transfer.

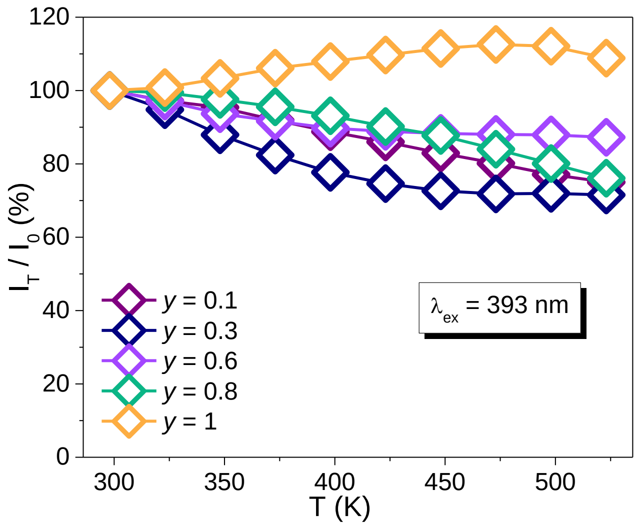

Figure S17. Relative integrated intensity ratio (I_T/I_0, I_T represents integrated intensity at temperature T, I_0 represents integrated intensity at temperature 298 K) *vs* heating temperature in MYG:yEu^{3+}, 0.01Mn^{4+} ($0.1 \leq y \leq 1$) monitored at λ_{ex} = 393 nm.

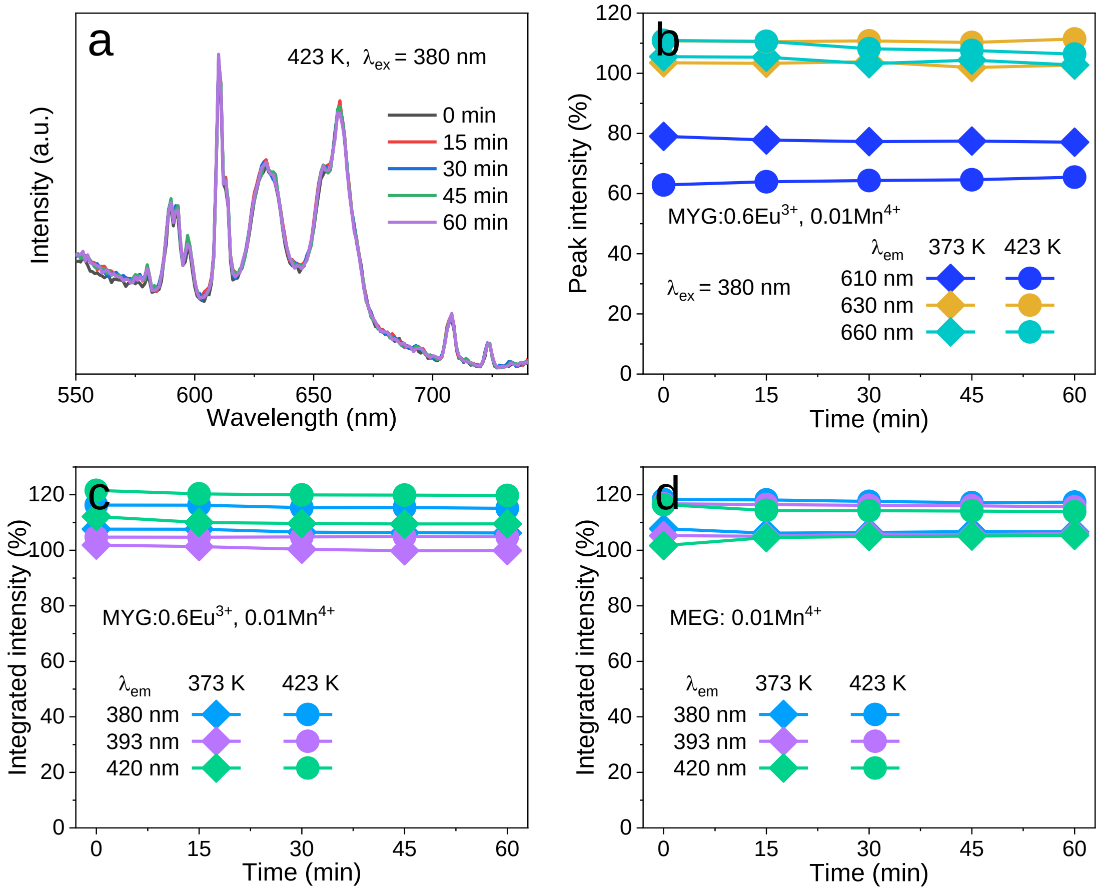

Figure S18. (a) The heating-time-dependent PL spectra of MYG:0.6Eu^{3+}, 0.01Mn^{4+} at 437 K with the excitation wavelength of 380 nm. (b) The peak intensity variation for 610 nm, 630 nm and 660 nm as function of heating time in MYG:0.6Eu^{3+}, 0.01Mn^{4+} (373 and 423 K). The relationship between integrated intensity and heating time under 380, 393 and 420 nm for (c) MYG:0.6Eu^{3+}, 0.01Mn^{4+} and (d) MEG:0.01Mn^{4+}.

Table S3. Optical parameters of several Mn^{4+}-doped thermometry sensors materials[3-8]

Samples	S_a (K^{-1})	S_r (%K^{-1})	Temperature range (K)
Y_2MgTiO_6:Mn^{4+}	–	1.42×10^{-3} [153 K]	10–513
$Cs_2WO_2F_4$:Mn^{4+}	–	0.21 [167 K]	
$SrAl_{12}O_{19}$:Mn^{4+}	4.17×10^{-3}	2.7×10^{-3} [393 K]	–
$Lu_3Al_5O_{12}$:Mn^{4+}	–	3.75 [311 K]	–
$Y_3Al_5O_{12}$:Eu^{3+}/Mn^{4+}	0.441	4.81	293–393
$Na_2WO_2F_4$:Mn^{4+}	–	0.658 [193 K]	
MYG:Mn^{4+} (this work)	5×10^{-3} [300 K]	0.014 [200 K]	7–300
MEG:Mn^{4+} (this work)	0.015 [175 K]	0.019 [300 K]	7–300

References

[1] X. Li, P. Li, Z. Wang, S. Liu, Q. Bao, X. Meng, K. Qiu, Y. Li, Z. Li, Z. Yang, *Chem. Mater.* **2017**, *29*, 8792.

[2] M. Zhao, Z. Xia, M. S. Molokeev, L. Ning, Q. Liu, *Chem. Mater.* **2017**, *29*, 6552.

[3] S.-H. Yang, Y.-C. Lee, Y.-C. Hung, *Ceram. Int.* **2018**, *44*, 11665.

[4] F. Li, J. Cai, F. Chi, Y. Chen, C. Duan, M. Yin, *Opt. Mater.* **2017**, *66*, 447.

[5] D. Chen, S. Liu, Y. Zhou, Z. Wan, P. Huang, Z. Jia, *J. Mater. Chem. C* **2016**, *4*, 9044.

[6] P. Cai, X. Wang, H. J. Seo, *Phys. Chem. Chem. Phys.* **2018**, *20*, 2028.

[7] P. Cai, L. Qin, C. Chen, J. Wang, S. Bi, S. I. Kim, Y. Huang, H. J. Seo, *Inorg. Chem.* **2018**, *57*, 3073.

[8] P. Cai, L. Qin, C. Chen, J. Wang, H. J. Seo, *Dalton Trans.* **2017**, *46*, 14331.
